# Antioxidant Potential, Genotoxic Safety, and Metabolomic Profiling of Cladode Extracts from *Dactylopius opuntiae*-Resistant *Opuntia* Species

**DOI:** 10.3390/antiox15040416

**Published:** 2026-03-26

**Authors:** Abderrahmane Hadini, Mounsef Neffa, Sanae Baddaoui, Mourad Bendada, Mohammadine Moumou, Amani Tayebi, Kaoutar Aboukhalid, Abdesselam Maatougui, Ennouamane Saalaoui, Maria D’Elia, Khalid El Bekkaye, Luca Rastrelli, Anthony Bernard, Hicham Harnafi

**Affiliations:** 1 Laboratory of Bioresources, Biotechnologies, Ethnopharmacology and Health, Faculty of Sciences, Mohammed First University, Oujda 60000, Moroccom.neffa@ump.ac.ma (M.N.);; 2 Laboratory of the Improvement of Agricultural Production, Biotechnology and the Environment, Faculty of Sciences, Mohammed First University, Oujda 60000, Morocco; 3 Plant Ecology Laboratory, Regional Center of Agricultural Research of Oujda, National Institute of Agricultural Research, Avenue Ennasr, BP 415 Rabat Principale, Rabat 10090, Morocco; 4 Dipartimento di Farmacia, University of Salerno, Via Giovanni Paolo II, 132, Fisciano, 84084 Salerno, Italy; mdelia@unisa.it; 5 National Biodiversity Future Center (NBFC), 90133 Palermo, Italy; 6 Dipartimento di Scienze Della Terra e del Mare, University of Palermo, 90133 Palermo, Italy; 7 INRAE, University of Bordeaux, Unité Mixte de Recherche (UMR), Biologie du Fruit et Pathologie (BFP), 33140 Villenave d’Ornon, France; anthony.bernard@inrae.fr

**Keywords:** *Opuntia species*, *Dactylopius opuntiae* resistance, cladode extracts, polyphenols, antioxidant capacity, genotoxic safety, UHPLC-HRMS metabolomics

## Abstract

Species of the *Opuntia* genus are widely recognized for their richness in bioactive metabolites and antioxidant potential, particularly in their cladodes. However, despite increasing interest in cochineal-resistant cultivars, their genotoxic safety remains poorly explored. In this study, the phytochemical composition, antioxidant activity, and genotoxic effects of cladode extracts from three *Dactylopius opuntiae*-resistant *Opuntia* species (*O. ficus-indica*, *O. robusta*, and *O. stricta*) collected in eastern Morocco were comparatively evaluated. Hydroethanolic extracts were characterized for their biochemical composition and screened for antioxidant activity using DPPH, β-carotene bleaching, FRAP, and total antioxidant capacity assays. An untargeted UHPLC-Orbitrap MS/MS approach was applied to profile secondary metabolites, while genotoxicity was assessed using the comet assay on rat leukocyte DNA. The three species exhibited distinct phytochemical and antioxidant profiles. *O. ficus-indica* showed the highest total phenolic and flavonoid contents and the strongest radical scavenging and reducing capacities, whereas *O. stricta* was particularly rich in ascorbic acid and exhibited the highest total antioxidant capacity. Metabolomic analysis revealed a predominance of phenolic acids and flavonoids, with piscidic acid as a major constituent, along with isorhamnetin derivatives and organic acids. Importantly, none of the extracts induced genotoxic effects compared to the negative control, while all differed significantly from the oxidative damage induced by hydrogen peroxide. Overall, these findings demonstrate the phytochemical richness, antioxidant potential, and genotoxic safety of cochineal-resistant *Opuntia* cladodes, supporting their sustainable valorization in food, nutraceutical, cosmetic, and agricultural applications.

## 1. Introduction

Plant species, numbering in the thousands, have been globally dispersed for various purposes such as agriculture, forestry, and decoration. Among these, the *Opuntia* cactus, with nearly 1500 varieties from the Cactaceae family, is predominantly found in Africa and Mediterranean regions; these cacti are unique for their ability to thrive in harsh environments where other flora cannot [1]. *Opuntia* spp. has increasingly been recognized in recent years for its significant economic value, attributed to its beneficial nutritional and health-related benefits, its promising role in the cosmetic industry, its environmental applications, and the high-value products it generates [2,3]. Across diverse regions in Morocco, various *Opuntia* species are cultivated [4,5]. In 2014, the *O. ficus indica* L. species, predominantly found in Morocco, was initially infested by the cochineal insect *Dactylopius opuntiae* in the Sidi Bennour region [6]. This emergence posed a significant threat to the *Opuntia* spp. particularly *O. ficus indica* L. species, compelling researchers to implement biological and chemical control strategies to mitigate the impact of this pest [7,8]. However, recent research in Morocco has identified eight varieties of cactus pear that exhibit resistance to the cochineal. These cultivars are presently included in the recognized catalog of species, presenting a promising opportunity for their cultivation and exploitation [9,10].

Beyond their agronomic relevance, cochineal-resistant *Opuntia* varieties represent an emerging biological resource whose chemical composition and biological safety still require systematic investigation, particularly in view of their large-scale valorization and human exposure.

Among the components of these *Opuntia* species that have been extensively studied, cladodes are noteworthy. They are the green stems of the plants, often used as feed for livestock [11]. Indeed, cladodes are a valuable biomass rich in high-value products such as mucilage, which serves as a sustainable source of premium hydrocolloid carbohydrates, well-suited for both food and industrial applications [12]. The cladodes of *Opuntia* spp. are packed with water, fiber, proteins, polysaccharides, as well as vitamins and minerals, along with bioactive secondary metabolites (SMs) such as phenolic acids, which contribute to their notable antioxidant and potential anti-cancer effects, further enhancing their nutritional value [13,14,15,16]. In this context, the antioxidant properties of *Opuntia* cladodes have attracted increasing attention, as oxidative stress plays a key role in the pathogenesis of chronic, inflammatory, and degenerative diseases.

Additionally, they showed a significant antimicrobial and anti-inflammatory activity, making them commonly used as a functional food and in traditional medicine [17]. In many studies, the extracts of cladodes from different *Opuntia* species have revealed the presence of various interesting bioactive compounds using LC-MS or LC-PDA. These include flavonoids such as quercetin and its derivatives, as well as kaempferol and its analogues. Other notable molecules identified include isorhamnetin derivatives, quinic acid and piscidic acid. Such compounds are of particular interest for their potential biological properties [18,19,20]. Another approach to assess the phytochemical richness of *Opuntia* cladodes is untargeted metabolomics using LC-MS/MS. This strategy is widely used and allows for the evaluation of complex plant samples, the identification of low-mass compounds, and a better understanding of the metabolic pathways involved. It also offers the possibility of characterising all metabolites present in plant extracts, thereby facilitating the discovery of new compounds and previously unknown metabolic pathways [21,22].

Within this framework, untargeted metabolomics represents a powerful tool not only for comprehensive chemical profiling, but also for supporting the interpretation of antioxidant responses through the identification of compound classes involved in redox-related mechanisms. Despite the extensive literature describing the antioxidant and protective effects of *Opuntia* cladode extracts, information regarding their potential genotoxic effects remains limited and fragmented, particularly for recently selected cochineal-resistant species intended for food, nutraceutical, or cosmetic use. On the other hand, previous research indicates that the extracts from prickly pear cladodes may provide protective effects against agents that induce DNA strand alterations and breaks [23,24], and LPS-induced toxicity by modulating cell cycle and apoptosis-related proteins [25]. This allows for the valorization of Prickly Pear cladodes across various domains, leveraging their potential as high-value-added products [18,26]. In this regard, the present study aims to evaluate the antioxidant activity and assess, for the first time, the genotoxicity of extracts from the cladodes of three species of cacti from the genus *Opuntia*, recently confirmed as resistant to the *D. opuntiae* cochineal, collected from the eastern region of Morocco, an area known for its semi-arid climate. In addition, an untargeted metabolomic approach was used to characterize the chemical composition of the extracts and to identify the main classes of metabolites that may be responsible for their biological activities. By integrating antioxidant assays, untargeted metabolomic profiling, and genotoxicity assessment, this study aims to provide a scientifically robust basis for the safe and sustainable exploitation of these *Opuntia* species. These species were selected for their significant adaptability across various climatic regions, in order to assess their safety, potential advantages and value in various applications.

## 2. Materials and Methods

### 2.1. Biochemical Analysis

All biochemical analyses were conducted in triplicate. The chemicals employed in this research are of analytical grade and were purchased from Sigma-Aldrich chemicals (St. Louis, MO, USA).

### 2.2. Preparation of Plant Material

The study utilized fresh cladodes from three species of *Opuntia*, which have been identified by the National Institute for Agricultural Research (INRA), as newly discovered species resistant to the cochineal pest *D. opuntiae*. Species identification was performed by INRA experts, and voucher specimens were archived at the INRA experimental station for reference. These species include *Opuntia ficus-indica* (L.) Mill, *Opuntia robusta J.C. Wendl*, and *Opuntia stricta (Haw.)* Haw. The samples were collected during May and June 2023 at the INRA experimental station in Bouareg, located in the Nador region of northeastern Morocco (35°5′18.12″ N, 02°55′33.35″ W). After collection, the cladodes were carefully stripped of their spines, rinsed with distilled water, and left to air-dry in a shaded area for a week. Air-drying was performed under controlled ambient conditions to minimize enzymatic degradation and preserve secondary metabolites. The pieces were then cut into smaller sections and dried again in a ventilated oven at 37 °C for 48 h. After drying, the material was ground using an electric grinder (model 0405 Silver, Moulinex, Écully, France) and sifted through a 120-mesh sieve (125 μm). The final powder was stored in airtight bags, kept away from light, until further use.

### 2.3. Measurement of Fiber Content

The assessment of fiber content was performed using an adapted Van Soest method [27], which allows for a comprehensive and direct measurement of hemicelluloses, cellulose, and lignin, using a fiber extractor (Fiwe 6 Velp Scientifica Srl, Usmate, Italy). The process involves three hydrolysis steps: neutral detergent fiber (NDF), acid detergent fiber (ADF), and acid detergent lignin (ADL). Approximately 1 g of each sample, ground and dried at 50 °C, was used. The samples were placed in crucibles after weighing, to which 100 mL of prepared NDF solution was added (composed of 60 g sodium lauryl sulfate, 37.22 g disodium EDTA, 13.62 g disodium tetraborate 10 H_2_O, and 9.12 g triethylene glycol, in 20 mL disodium hydrogenophosphate solution in 2 L of distilled water), The crucibles were left in the extractor at 70 °C for 1 h. Once the process was completed, the crucibles were placed in an oven to dry at 110 °C for 24 h. Subsequently, the NDF weight was assessed. The operating procedure for ADF is practically the same as for NDF, only the detergent solution changes. Approximately 56 mL of 98% sulfuric acid (H_2_SO_4_) with 40 g of cetyltrimethylammonium bromide (CTAB) powder in 2 L of distilled water is prepared as the ADF detergent solution. The ADL solution is prepared using 72% H_2_SO_4_, the crucibles are filled and left for 3 h, rinsed in the extractor, then incubated in the oven at 110 ± 1 °C for 24 h. After 48 h, the weight of ADL was determined, and finally, the crucibles were incinerated in a muffle furnace at 550 °C for 3 h to determine the incineration weight. To calculate the percentages of hemicellulose, cellulose, and lignin, the following Equations (1)–(6) are used:

(1) Hemicellulose (%) = NDF − ADF

(2) Cellulose (%) = ADF − ADL

(3) Lignin (%) = ADL

(4) NDF (%) = (W1 − W4/E) × 100

(5) ADF (%) = (W2 − W4/E) × 100

(6) ADL (%) = (W3 − W4/E) × 100

W0 (g): weight of the empty crucible (tare)
W1 (g) = NDF: weight of crucible + residue after NDF treatment (cellulose + hemicellulose + lignin + ash)
W2 (g) = weight of crucible + residue after ADF treatment (cellulose + lignin + ash)
W3 (g) = weight of crucible + residue after ADL treatment (lignin + ash)
W4 (g) = weight of crucible + ash after incineration
E (g) = initial sample weight.

### 2.4. Photosynthetic Pigments

Photosynthetic pigment analysis was performed by finely grinding about 0.2 g of fresh cladode tissue in 20 mL of 80% acetone solution. The resulting mixture was then kept in the dark at ambient temperature for a duration of 48 h [28]. Absorbance was measured using a UV–Vis spectrophotometer (Shimadzu UV-1800, Kyoto, Japan), at wavelengths of 663, 645, and 652 nm. The contents of chlorophyll a, chlorophyll b, and total chlorophyll were calculated based on the given Equations (7)–(9):

(7) Chl a (mg/g) = (12.21 × Abs663) − (2.81 × Abs645)

(8) Chl b (mg/g) = (20.13 × Abs645) − (5.03 × Abs663)

(9) Total Chl = Chl a + Chl b

### 2.5. Ascorbic Acid Concentration

The ascorbic acid level was assessed using a colorimetric method based on the modified procedure described by Mau [29]. Approximately 1 g of dried sample powder from each species was combined with 10 mL of 1% oxalic acid and stirred for 15 min. The resulting solution was filtered, and 3 mL of the filtrate was then mixed with 1 mL of a 5 mM aqueous solution of 2,6-dichlorophenolindophenol (DCPIP). After 15 s, the absorbance was recorded at a wavelength of 515 nm. The results were calculated as milligrams of ascorbic acid equivalent per 100 g of dry matter (mg AscAE/100g DM).

### 2.6. Preparation of Cladode Hydroethanolic Extracts

The extraction was performed using an ethanol/water mixture (70/30, *v*/*v*) for 24 h with continuous stirring in the dark at room temperature. 2 g of cladodes from each of three *Opuntia* species were combined with 50 mL of the solvent. After maceration, the mixture was filtered, and the solvent was removed under reduced pressure using a rotary evaporator (Buchi Waterbath B-480, Flawil, Switzerland) at 40 °C. The dry extract was stored at −20 °C until needed and the extraction was calculated as a percentage (%). The hydroethanolic system was selected to efficiently extract a broad range of polar and semi-polar metabolites.

#### 2.6.1. Reducing Sugar Content Determination

The determination of reducing sugars was carried out using the 3,5-dinitrosalicylic acid (DNSA) method [30], with minor modifications. 0.5 g of DNS and 15 g of sodium-potassium tartaric acid were dissolved in 40 mL of 0.5N NaOH and incubated at 45 °C until dissolution, then allowed to cool to room temperature. 0.5 mL of the hydroethanolic extract of cladodes was used by adding 0.5 mL of DNS reagent and placing the mixture in a water bath at 100 °C for 5 min. The tubes were then removed and placed in cold water. Next, 5 mL of distilled water was added, and the absorbance was measured at 540 nm using a spectrophotometer Biotek Gen5 microplate reader (BioTek Gen5, Winooski, VT, USA). The calculation of reducing sugars was carried out referring to a calibration curve obtained using D-glucose (mg D-gluE/mL).

#### 2.6.2. Total Polyphenols

The determination of total phenolic content (TPC) was performed using the Folin–Ciocalteu method [31]. For each sample, 500 μL of diluted cladode extract was mixed with 3.75 mL of distilled water, followed by the addition of 250 μL of Folin–Ciocalteu reagent. After 5 min, 0.5 mL of a 20% (*w*/*v*) sodium carbonate solution was added, and the mixture was incubated in the dark at room temperature for 30 min. Absorbance was measured at 760 nm, and results were expressed as milligrams of gallic acid equivalents per g of dry matter (mg GAE/g DM).

#### 2.6.3. Flavonoid Content

Total flavonoid content (TFC) was determined using a colorimetric method based on aluminum chloride complexation [32]. Briefly, 500 μL of diluted cladode extract was mixed with 500 μL of a 2% (*w*/*v*) AlCl_3_ solution in methanol. The mixture was incubated in the dark at room temperature for 40 min, after which absorbance was measured at 430 nm. Results were expressed as milligrams of rutin equivalents per g of dry matter (mg RE/g DM).

#### 2.6.4. Condensed Tannins Content

Condensed tannin content was determined using the vanillin assay according to the protocol described by Julkunen [33]. An aliquot of 50 μL of extract was mixed with 1500 μL of a 4% (*w*/*v*) vanillin solution in methanol, followed by the addition of 750 μL of concentrated HCl. The reaction mixture was allowed to stand at room temperature for 20 min, and absorbance was recorded at 550 nm. Condensed tannin content was expressed as milligrams of cyanidin equivalents per g of dry matter (mg CE/g DM).

### 2.7. In Vitro Antioxidant Activity

#### 2.7.1. DPPH Free Radical Neutralization

To assess the DPPH free radical scavenging activity of cladode extracts, solutions at concentrations ranging from 0.5 to 4 mg/mL were prepared. Antioxidant activity was evaluated using a method modified from Braca [34]. Briefly, 0.1 mL of cladode extract was mixed with 2.9 mL of a methanolic DPPH solution (6 × 10^−5^ M). The mixture was incubated in the dark at room temperature for 30 min, and absorbance was measured at 517 nm. The control consisted of distilled water instead of the extract. The percentage of DPPH radical scavenging activity was calculated using the following Equation (10):

(10) (%) Inhibition activity (DPPH) = ((Abs control − Abs samples))/Abs control × 100

#### 2.7.2. β-Carotene Bleaching Assay

The β-carotene degradation assay was performed based on the method described by Kabouche et al. [35]. Briefly, 4 mg of β-carotene were initially dissolved in 2 mL of chloroform, followed by the addition of 40 mg of linoleic acid and 400 mg of Tween 80. After evaporation of chloroform under vacuum, 100 mL of distilled water was added to the residue, and the mixture was vigorously vortexed to form an emulsion. Subsequently, 100 μL of each extract, at a fixed concentration of 5 mg/mL, was added to test tubes containing 2.45 mL of the β-carotene/linoleic acid emulsion. The initial absorbance (Ai) was measured at 470 nm, followed by incubation at 50 °C for 2 h, after which the final absorbance (Af) was recorded. Methanol was used as the negative control. The extent of β-carotene bleaching was calculated using the following Equation (11):

(11) Proportion of β-carotene degradation: [(Ai − Af)/Ai] × 100

#### 2.7.3. Ferric Ion Reduction Antioxidant Assay

The ferric reducing antioxidant power (FRAP) of cladode extracts was determined using a modified method described by Karagözler et al. [36]. Extracts were diluted in methanol to the desired concentrations. An aliquot of 1 mL of each dilution was mixed with 2.5 mL of phosphate buffer (0.2 M, pH 6.6) and 2.5 mL of a 1% (*w*/*v*) potassium ferricyanide (K_3_Fe(CN)_6_) solution. The mixture was incubated at 50 °C for 20 min and then cooled to room temperature. The reaction was stopped by adding 2.5 mL of 10% (*w*/*v*) trichloroacetic acid, followed by centrifugation at 3000 rpm for 30 min. Subsequently, 2.5 mL of the supernatant was mixed with 2.5 mL of distilled water and 500 μL of a freshly prepared 0.1% (*w*/*v*) FeCl_3_ solution. Absorbance was measured at 700 nm using distilled water as the blank. Results were expressed as milligrams of α-tocopherol equivalents per g of dry matter (mg α-TocE/g DM).

#### 2.7.4. Total Antioxidant Capacity

A Phosphomolybdate Assay (TAC) was performed according to the protocol of Umamaheswari and Chatterjee [37]. To determine the total antioxidant capacity, 100 μL of the extract (1 mg/mL) was combined with 1 mL of a reagent solution containing 0.6 M sulfuric acid, 28 mM sodium phosphate, and 4 mM ammonium molybdate. This mixture was then heated in a water bath at 95 °C for 90 min. After incubation, the absorbance was recorded at 695 nm. Antioxidant levels were reported as milligrams of α-tocopherol equivalents per g of dry matter (mg α-TocE/g DM).

### 2.8. UHPLC-Orbitrap IQ-X Tribrid MS/MS Analysis of Cladodes Extracts

Dried extracts of cladodes (10 mg of dry matter) were resuspended in 500 µL of hydroethanolic solution (ethanol/water, 70:30, *v*/*v*). The extracts were homogenized by vortexing for 30 s and manually filtered using Millex™ nylon syringe filters (33 mm, 0.45 µm, hydrophilic, non-sterile; Merck KGaA, Darmstadt, Germany) mounted on 1 mL syringes. Filtered extracts were collected in Micronic microtubes (Micronic B.V., Lelystad, The Netherlands). A pooled quality control (QC) sample was prepared by combining 50 µL of each filtered extract into a single Micronic tube and vortexing. The pooled QC was used to assess system stability and method reproducibility. Chromatographic separation was carried out on a Gemini C18 column (Phenomenex, Torrance, CA, USA) operated in reverse-phase mode at 40 °C with a flow rate of 0.350 mL/min. The injection volume was 5 µL. The mobile phase consisted of solvent A (ultrapure Millipore water with 0.1% formic acid) and solvent B (acetonitrile with 0.1% formic acid). The elution gradient was as follows: 0.0 min, 3% B; 1.0 min, 10% B; 9.0 min, 50% B; 13.0 min, 100% B; 14.5 min, 3% B, with a total run time of 18 min, followed by re-equilibration at 3% B. Mass spectrometry was performed using an Orbitrap IQ-X Tribrid mass spectrometer (Thermo Fisher Scientific, Bremen, Germany) equipped with an electrospray ionization (ESI) source. Analyses were conducted in negative mode with an ionization voltage of 2500 V, vaporizer temperature of 300 °C, and ion transfer tube temperature of 300 °C. Full-scan MS (MS1) was performed at a resolution of 90,000 with a scan range of *m*/*z* 70–1050, using internal calibration with EASY-IC. Data-dependent MS/MS (DDA) analyses were performed at a resolution of 30,000 using high-energy collision dissociation (HCD) with a normalized collision energy of 25% and a maximum injection time of 54 ms. The analytical sequence included blank injections (pure solvent) at the beginning and end of the run. A pooled QC was injected at the start to stabilize the system, followed by randomly ordered sample injections to minimize instrumental drift. The QC pool was re-injected every 6–10 sample injections to monitor system stability and reproducibility. Data quality was assessed by evaluating retention time variation (<±0.2 min) and signal intensity reproducibility for major compounds (CV < 15%). Metabolite identification was performed by comparing accurate mass measurements and MS/MS fragmentation patterns with entries in the PubChem and METLIN databases, using SIRIUS software (version 6.2.2) and consulting the relevant literature. Relative peak areas were used for qualitative comparison of the chemical composition among the different extracts. All LC-MS analyses were performed at the Bordeaux Metabolome Platform (MetaboHUB, Bordeaux, France).

### 2.9. Genotoxicity Evaluation of Opuntia Cladode Extracts

#### 2.9.1. Animals and Experimental Conditions

To evaluate the genotoxicity of *Opuntia* cladode extracts, 70% ethanol was used for extraction, given its recognition by the FDA as a Generally Recognized as Safe (GRAS) designation, indicating endorsement by experts with specialized qualifications for its safety and suitability in food products [38]. We used blood cells from male Wistar rats, 6 to 8 weeks old and ranging in weight from 150 to 200 g. The rats were kept in the research facilities of the Faculty of Sciences, Oujda, Morocco. The ambient temperature was kept constant between 19 and 23 °C. They had unlimited access to both food and water. Rat care and handling complied with international guidelines for the care and use of laboratory animals.

#### 2.9.2. Comet Electrophoresis Assay

The comet assay was performed as described by Singh et al. [39], and adapted by Ouahhoud et al. with slight modifications [38]. A leukocyte suspension was first incubated for 5 min and then subjected to centrifugation at 4500 rpm for 10 min. The pellet, which contained leukocytes, was dissolved in 1 mL of PBS while the supernatant was discarded. The washing procedure was repeated three times. Then, the leukocyte suspension was incubated with ethanolic extracts of *Opuntia* cladodes at four concentrations (10, 50, 100, and 200 µg/mL) for 15 min at 37 °C. As a positive control, 200 µL of H_2_O_2_ solution (250 µM) was added to the medium, while for the negative control, an additional 200 µL of PBS (pH 7.4, free of Ca^2+^ and Mg^2+^) was used under the same conditions. The pellet was then resuspended in 200 μL of 0.5% *w*/*v* low-melting-point agarose in PBS, and the mixture was applied onto a slide that had been pre-coated with 1.5% *w*/*v* normal melting-point agarose in PBS. The slides were subsequently incubated for 1 h in the lysis solution (containing 2.5 M NaCl, 100 mM Na_2_EDTA, 20 mM Tris, 300 mM NaOH, 1% sodium N-lauroylsarcosinate, 10% DMSO, and 1% Triton X-100) at 4 °C, shielded from light. Next, the slides were carefully rinsed with distilled water and placed horizontally in the electrophoresis chamber, which was filled with freshly made electrophoresis buffer (300 mM sodium hydroxide and 1 mM Na_2_EDTA, pH 13). For 20 min, the DNA was permitted to unwind, then migration proceeded for 20 min at a constant voltage of 20 V and a current intensity of 300 mA. The electrophoresis solution temperature was kept at 4 °C throughout the running and electrophoresis process. The slides were submerged in a buffer solution (400 mM Trizma, pH 7.5 adjusted with HCl) for 5 min to neutralize. After electrophoresis and neutralization, the comets were examined using a silver staining technique as described by Garcia et al. [40]. They were soaked in a fixative solution (15% trichloroacetic acid, 5% zinc sulfate, and 5% glycerol) for 10 min, then rinsed with distilled water. To color the slides, a solution composed of 32 mL of sodium carbonate (5%) and 68 mL of a mixture containing ammonium nitrate (0.02%), silver nitrate (0.02%), tungstosilicic acid (0.1%), and formaldehyde (0.05%) was carefully applied to the slides placed in a slide box. This step was performed in complete darkness at ambient temperature. Then, the slides were immersed in a neutralizing solution of 1% acetic acid for 5 min. Finally, the slides were thoroughly washed with distilled water and left to dry naturally at room temperature.

#### 2.9.3. Microscopic Observation

The slides stained with silver nitrate were examined under a ZOE Fluorescent Cell Imager microscope (Bio-Rad, Hercules, CA, USA), with a 400× objective [40]. Capturing of images was conducted using a CMEX 5000 camera (Pioneer, CA, USA), and analysis was carried out using CaspLab software (v1.2.3 beta 1). Quantitative assessment of DNA damage was facilitated through image analysis software, CaspLab, allowing for the measurement of different indicators related to DNA damage. Each sample was analyzed in duplicate, with fifty randomly selected cells per replicate.

### 2.10. Statistical Analysis

Data analysis was performed using R software (version 4.3.2). Data normality was assessed using the Shapiro–Wilk test. Homogeneity of variances and homoscedasticity were evaluated using Levene’s and Bartlett’s tests, respectively. Differences among groups were analyzed by one-way analysis of variance (ANOVA), followed by the Student–Newman–Keuls (SNK) post hoc test to identify statistically significant pairwise differences. Pearson and Spearman correlation coefficients were calculated to investigate relationships between biochemical parameters and antioxidant activities. In addition, principal component analysis (PCA) was applied to reduce data dimensionality and to visualize patterns and clustering among the biochemical variables of the three *Opuntia* species. Statistical significance was set at *p* < 0.05.

## 3. Results

The present study evaluated the antioxidant activity, metabolomic profile, and genotoxic potential of cladode extracts using a hydroethanolic method, resulting in yields of 12% for *O. ficus-indica*, 7.55% for *O. robusta*, and 6.12% for *O. stricta*. The extracts were obtained from the previous species selected for their resistance to biotic stress, particularly the cochineal *D. opuntiae*, and for their adaptation to arid and semi-arid bioclimatic conditions. These characteristics make cochineal-resistant *Opuntia* species promising candidates for sustainable agricultural systems under current climate change scenarios. Analysis of biochemical parameters revealed significant differences among the three species.

### 3.1. Dietary Fiber Content

The dietary fiber composition of the cladodes differed significantly among the three *Opuntia* species (Table 1). The highest neutral detergent fiber (NDF) content was observed in *O. robusta* (36.23%), followed by *O. ficus-indica* (32.94%), while *O. stricta* showed the lowest value (22.62%). Acid detergent fiber (ADF) content also varied significantly, with the highest level detected in *O. ficus-indica* (16.78%), whereas *O. robusta* and *O. stricta* exhibited lower ADF values (10.56% and 6.74%, respectively).

Acid detergent lignin (ADL) content ranged from 0.95% to 5.67%, with overall low lignin levels across all species. The highest ADL value was recorded in *O. ficus-indica*, while *O. stricta* showed the lowest lignin content. Cellulose content was significantly higher in *O. ficus-indica* (11.12%), whereas hemicellulose content reached its maximum in *O. robusta* (25.67%). All differences were statistically significant (*p* < 0.05).

### 3.2. Photosynthetic Pigment

**Figure 1:**
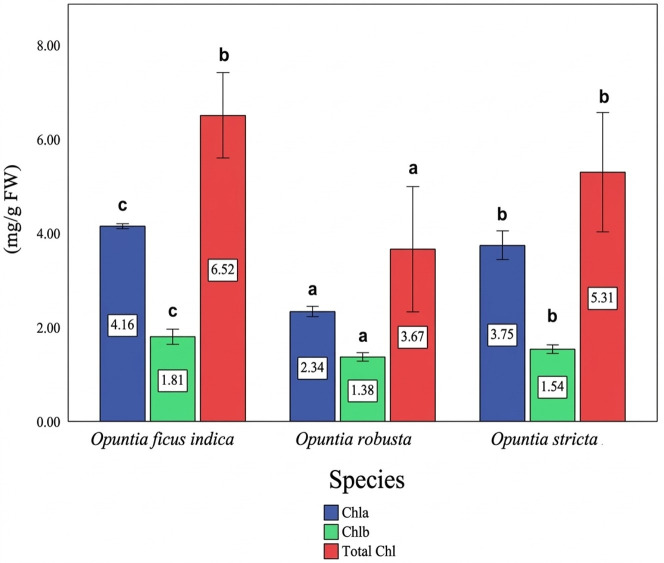
Chlorophyll a (Chl a), chlorophyll b (Chl b), and total chlorophyll contents in the cladodes of *Opuntia* species. Values represent the mean ± SD of three independent measurements. Different letters above the bars indicate statistically significant differences (*p* < 0.05) according to the Student–Newman–Keuls (SNK) test.

The cladodes of *Opuntia* species exhibited significant differences (*p* < 0.05) in chlorophyll a (Chl a), chlorophyll b (Chl b), and consequently total chlorophyll content (Figure 1). *O. ficus-indica* showed the highest chlorophyll levels, particularly for Chl a, reaching 4.16 mg/g fresh weight (FW). Intermediate chlorophyll contents were observed in *O. stricta* cladodes, with a Chl a value of 3.75 mg/g FW, whereas *O. robusta* exhibited the lowest concentrations for all chlorophyll fractions. Differences among species were statistically significant for all measured pigments.

### 3.3. Ascorbic Acid Content

**Figure 2:**
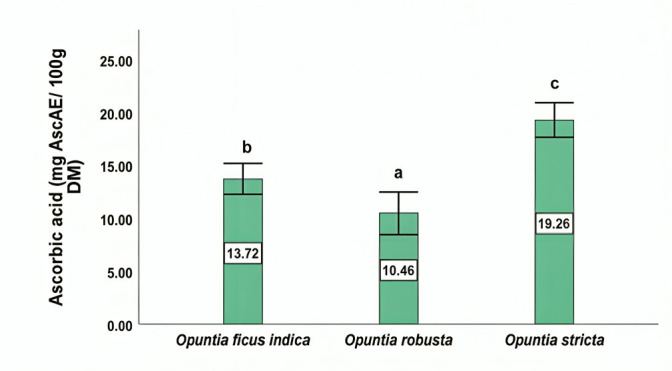
Ascorbic acid content in the cladodes of *Opuntia* species. Values are expressed as mean ± SD (*n* = 3). Different letters above the bars indicate statistically significant differences (*p* < 0.05) according to the Student–Newman–Keuls (SNK) test. AscAE, ascorbic acid equivalents; DM, dry matter.

The cladodes of *O. stricta* exhibited the highest ascorbic acid content (19.26 mg AscAE/100 g DM), significantly exceeding the levels measured in *O. ficus-indica* (13.72 mg AscAE/100 g DM) and *O. robusta* (10.46 mg AscAE/100 g DM) (Figure 2). Statistically significant differences among species were observed (*p* < 0.05).

### 3.4. Reducing Sugar Content

**Figure 3:**
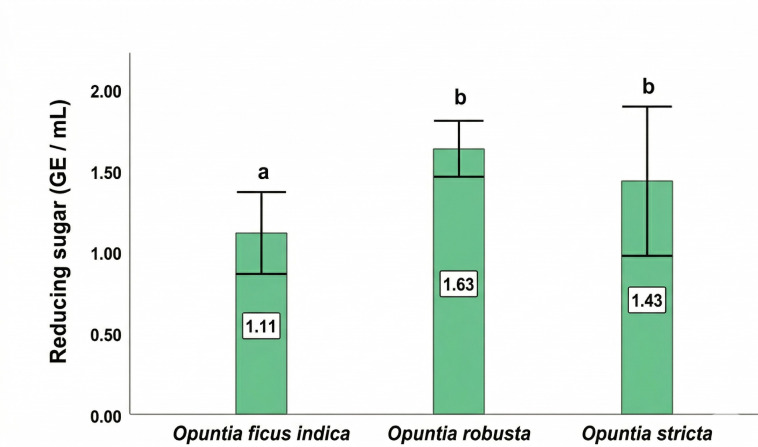
Reducing sugar content in the cladodes of *Opuntia* species. Values are expressed as mean ± SD (*n* = 3). Different letters above the bars indicate statistically significant differences (*p* < 0.05) according to the SNK test. GE, glucose equivalents.

The cladodes of the three *Opuntia* species showed significant differences (*p* < 0.05) in reducing sugar content (Figure 3). *O. robusta* exhibited the highest concentration of reducing sugars (1.63 ± 0.10 mg GE/mL), whereas the lowest value was recorded in *O. ficus-indica* cladodes (1.11 ± 0.08 mg GE/mL). *O. stricta* showed intermediate reducing sugar levels. Differences among species were statistically significant.

### 3.5. The Total Phenolic, Flavonoid, and Condensed Tannin Content

**Figure 4:**
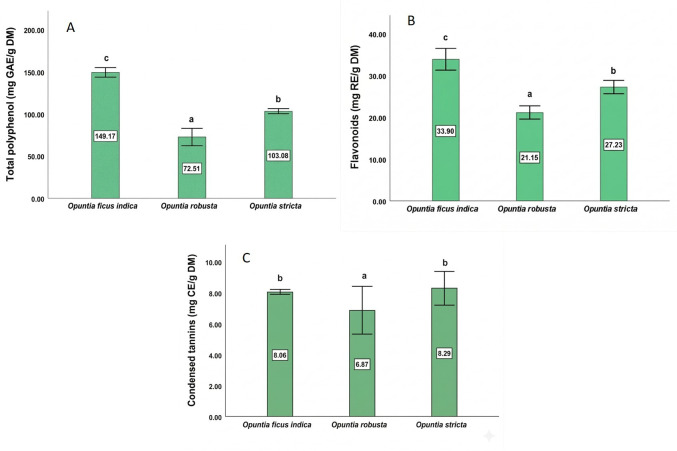
Total phenolic content (**A**), flavonoid content (**B**), and condensed tannin content (**C**) in the cladodes of *Opuntia* species. Values are expressed as mean ± SD (*n* = 3). Different letters indicate statistically significant differences (*p* < 0.05) according to the SNK test. GAE, gallic acid equivalents; RE, rutin equivalents; CE, cyanidin equivalents; DM, dry matter.

The contents of phenolic compounds, flavonoids, and condensed tannins differed significantly among the hydroethanolic cladode extracts (Figure 4). *O. ficus-indica* exhibited the highest total phenolic content (149.17 mg GAE/g DM) and flavonoid content (33.90 mg RE/g DM). In contrast, *O. stricta* showed the highest condensed tannin content (8.29 mg CE/g DM). *O. robusta* displayed the lowest levels for all three classes of secondary metabolites, with total phenolics of 72.51 mg GAE/g DM, flavonoids of 21.15 mg RE/g DM, and condensed tannins of 6.87 mg CE/g DM. Differences among species were statistically significant (*p* < 0.05).

### 3.6. Antioxidant Activity Assessed In Vitro

The antioxidant activities of the cladode extracts varied significantly among the three *Opuntia* species, as assessed by multiple in vitro assays (Table 2). In the DPPH radical scavenging assay, *O. ficus-indica* showed the strongest activity, with an IC_50_ value of 1.99 ± 0.89 mg/mL, followed by *O. stricta* (2.07 ± 0.91 mg/mL), whereas *O. robusta* exhibited the lowest activity.

A similar trend was observed in the β-carotene bleaching assay, where *O. ficus-indica* displayed the highest inhibition of β-carotene oxidation (48.52 ± 0.50%), while *O. robusta* and *O. stricta* showed lower inhibition values of 28.37 ± 1.18% and 25.46 ± 0.34%, respectively. In the FRAP assay, *O. ficus-indica* again demonstrated the highest reducing power (19.48 ± 0.94 mg α-TocE/g DM). Conversely, total antioxidant capacity (TAC) was highest in *O. stricta*, reaching 90.29 ± 1.34 mg α-TocE/g DM. All differences among species were statistically significant (*p* < 0.05).

### 3.7. Correlation Analysis and Principal Component Analysis

**Figure 5:**
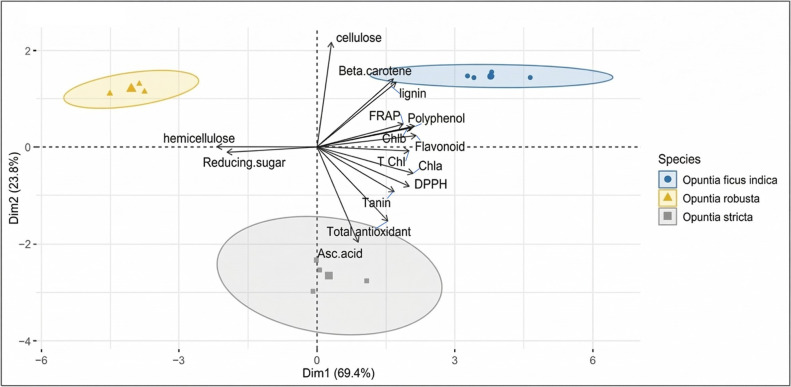
Principal component analysis (PCA) of phytochemical parameters and antioxidant activities in ethanolic extracts of *Opuntia* cladodes.

The relationships between phytochemical contents and antioxidant activities were further explored by Pearson correlation analysis and multivariate statistical analysis. Pearson correlation coefficients between total phenolic content (TPC), total flavonoid content (TFC), ascorbic acid, condensed tannins, and antioxidant assays (DPPH, FRAP, β-carotene bleaching, and total antioxidant capacity) are reported in Table 3. Several significant correlations were observed, highlighting strong associations between phenolic-related parameters and antioxidant activities. To provide an integrated overview of the relationships among biochemical variables and to visualize sample clustering, principal component analysis (PCA) was performed on the combined dataset. The PCA biplot (Figure 5) illustrates the distribution of *Opuntia* species according to their phytochemical composition and antioxidant profiles, as well as the contribution of individual variables to sample discrimination.

### 3.8. Comprehensive Metabolite Profiling of Opuntia Cladode Extracts Using UHPLC-Orbitrap IQ-X Tribrid MS/MS

In this study, an untargeted metabolomics approach based on UHPLC–Orbitrap IQ-X Tribrid MS/MS was used to characterize metabolites in cladode extracts of *O. ficus-indica*, *O. robusta*, and *O. stricta*. Annotated compounds were characterized based on retention times (RT), experimental *m*/*z* values acquired in negative ion mode ([M − H]^−^), proposed molecular formulas, and MS/MS fragmentation patterns, and were compared with data from the literature and metabolomics databases. Metabolite annotation was reported according to confidence levels, including Level 1 for compounds confirmed using reference standards, Level 2 for probable annotations supported by MS/MS data and database/literature matching, and Level 3 for tentative candidates proposed by SIRIUS software. Overall, 126 metabolites were annotated and grouped into major chemical classes (Table 4).

Phenolic acids and derivatives represented a major class (22 metabolites), including piscidic acid, caffeic acid, ferulic acid, *p*-coumaric acid, and sinapic acid, as well as several glycosylated and glucuronidated forms. These compounds were detected in all three species, with differences in peak area. Flavonoids were also prominent (32 metabolites), mainly occurring as glycosylated derivatives. Annotated flavonoids included derivatives of quercetin, kaempferol, isorhamnetin, myricetin, and apigenin, such as quercetin-3-*O*-rutinoside, isorhamnetin-3-*O*-rutinoside, astragalin, and several rutinosides, gentiobiosides, and sulfates.

In addition, the analysis enabled the annotation of organic acids, oxidized fatty acids (oxylipins), amino acids, and other metabolite classes. Major organic acids, including malic, citric, fumaric, gluconic, and quinic acids, were detected at high relative abundances. Several oxylipins, including 9-HODE, 13-Oxo-ODE, Me-9-HPODE, and 13S-HpOTrE, were also annotated. Among lipid-related features, relatively abundant signals included LPE(2:0) and PG O-11:0_24:4, together with other phospholipids. Free amino acids, including glycine, serine, proline, alanine, leucine, tyrosine, and histidine, were also detected. Additional annotated metabolites included sugars, polyols, nucleosides, and alkaloids.

**Figure 6:**
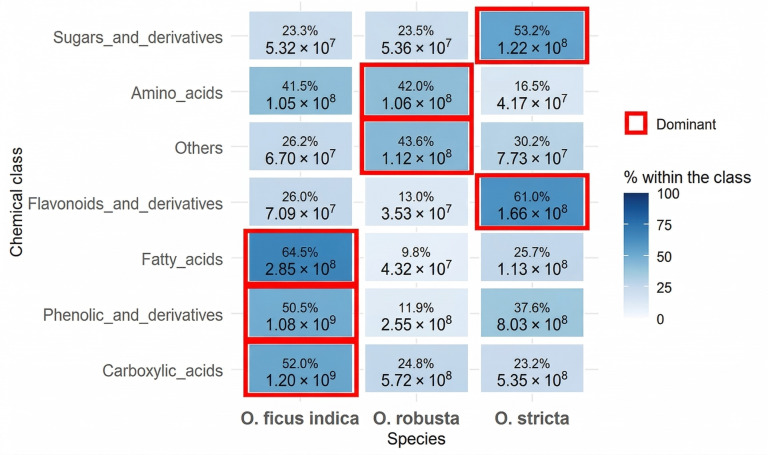
Heatmap showing the relative abundance patterns of annotated metabolite classes in cladode extracts of *Opuntia* species.

Overall, the three *Opuntia* species displayed distinct metabolite profiles (Figure 6). *O. ficus-indica* showed higher relative abundances of lipid-related features, phenolic compounds, and carboxylic acids, whereas *O. robusta* showed higher relative abundances of amino acids and alkaloid-related features. *O. stricta* exhibited higher relative abundances of flavonoids and sugars.

**Figure 7:**
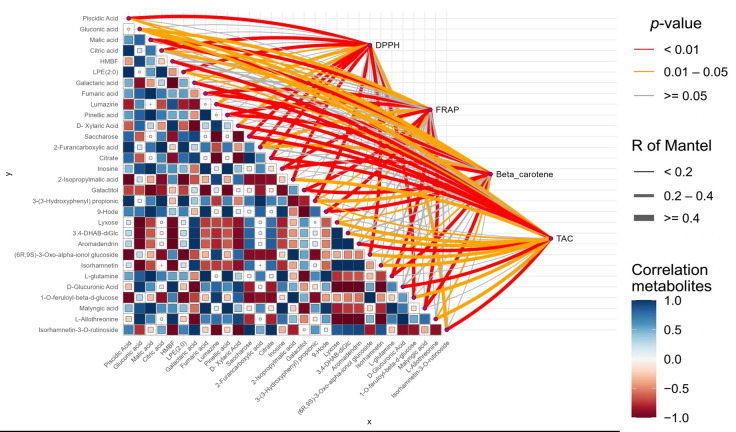
An integrated heatmap and Mantel test network illustrating the connections between antioxidant activity and the top 30 annotated metabolites. The heatmap, which ranges in color from −1 to 1, shows pairwise Pearson’s correlation coefficients (r). Network edges represent Mantel test results, where edge width indicates the Mantel’s R statistic and edge color reflects statistical significance (*p*-value). HMBF: 4,6-dihydroxy-3-(1-hydroxyethyl)-5-methoxy-3H-2-benzofuran-1-one.

**Figure 8:**
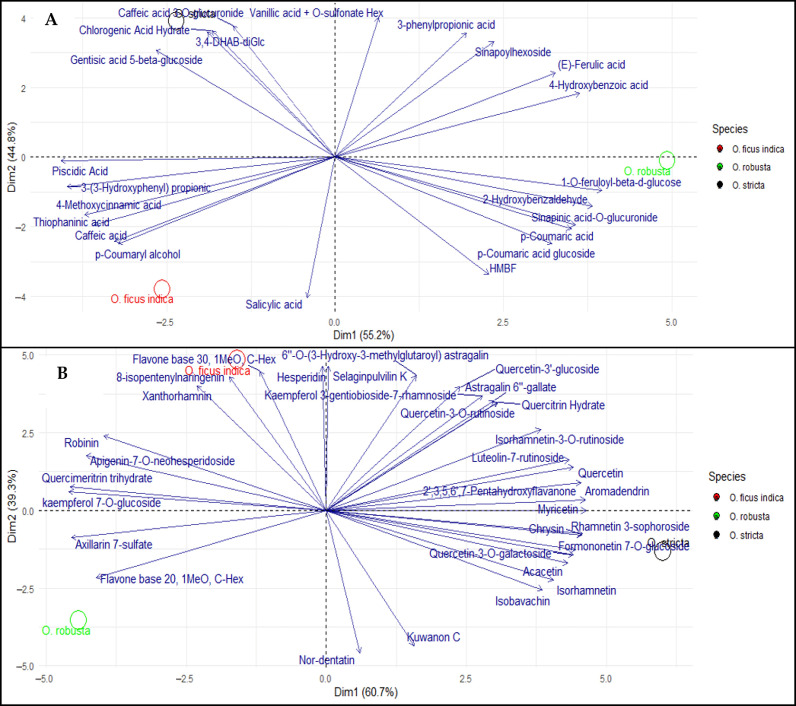
Principal Component Analysis (PCA) of the phenolic profiles of cladode extracts from *Opuntia* species. Biplots based on (**A**) phenolic acids and their derivatives and (**B**) flavonoids and their derivatives. Colors indicate species: *O. ficus-indica* (red), *O. robusta* (green), and *O. stricta* (black).

The top 30 metabolites were examined using a combined heatmap and Mantel test to investigate intra-metabolite correlations and their associations with antioxidant indicators, DPPH, FRAP, β-carotene, and TAC, in order to strengthen understanding and investigate the relationship between the major metabolites and the antioxidant activity (Figure 7). The correlation matrix highlights strong positive interactions among organic acids (malic, citric, fumaric) and flavonoids such as isorhamnetin, suggesting shared biosynthetic pathways or coordinated accumulation. The Mantel test network shows that most links to DPPH, FRAP, and TAC are highly significant (*p* < 0.01, R ≥ 0.4), indicating that phenolic acids and flavonoids (e.g., isorhamnetin-3-*O*-rutinoside, aromadendrin) are major contributors to TAC. In contrast, the β-carotene network displays fewer and weaker significant associations (0.01 ≤ *p* < 0.05), suggesting a more selective or indirect contribution. Key metabolites such as piscidic acid, isorhamnetin, and sugar derivatives (galactitol, sucrose) show multiple significant connections, confirming their central role. Overall, antioxidant activity arises from the synergistic contribution of a broad spectrum of metabolites rather than a single compound, highlighting the integrated nature of the metabolic network underlying the observed bioactivity. Additionally, phenolic acids and flavonoids generated from LC-MS analyses of cladode extracts were specifically grouped using a PCA. Strong correlations between the compounds and the various *Opuntia* species were found in the analysis (Figure 8), which highlighted unique chemical signatures for each species. *O. ficus-indica* is mainly linked to phenolic acids, while *O. stricta* is rich in flavonoids, especially glycosylated derivatives. These findings imply that both species significantly contribute to antioxidant activity, whereas *O. robusta* has a more distinct phenolic profile. These phenolic compounds’ abundance and variety highlight *Opuntia* cladodes’ significant bioactive potential.

### 3.9. Genotoxic Effect on Rat Leukocytes

**Figure 9:**
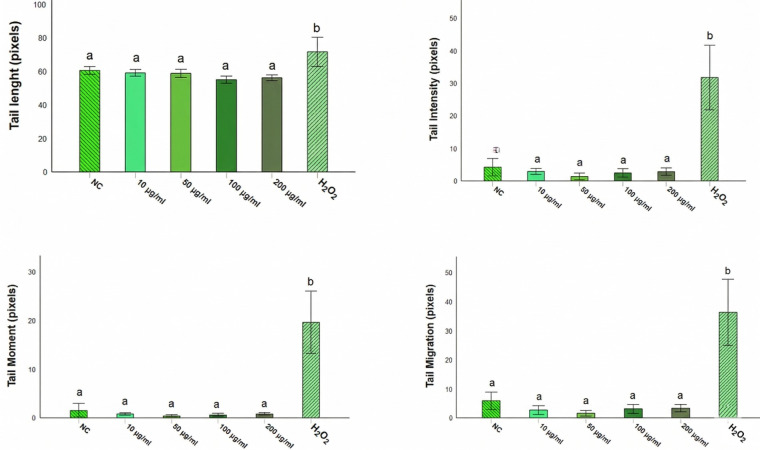
Comet assay parameters in rat leukocytes treated with *O. ficus-indica* cladode extract at different concentrations. Tail length (Tl), tail intensity (TI), tail moment (Tm), and tail migration (Tmi) are reported as mean ± SD. Different letters indicate statistically significant differences (*p* < 0.05) according to the Student–Newman–Keuls (SNK) test. NC, negative control (PBS); H_2_O_2_ (250 µM), positive control.

**Figure 10:**
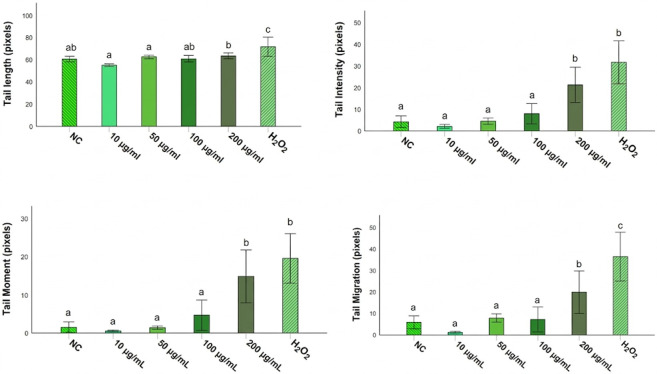
Comet assay parameters in rat leukocytes treated with *O. robusta* cladode extract at different concentrations. Tail length (Tl), tail intensity (TI), tail moment (Tm), and tail migration (Tmi) are reported as mean ± SD. Different letters indicate statistically significant differences (*p* < 0.05) according to the SNK test. NC, negative control (PBS); H_2_O_2_ (250 µM), positive control.

**Figure 11:**
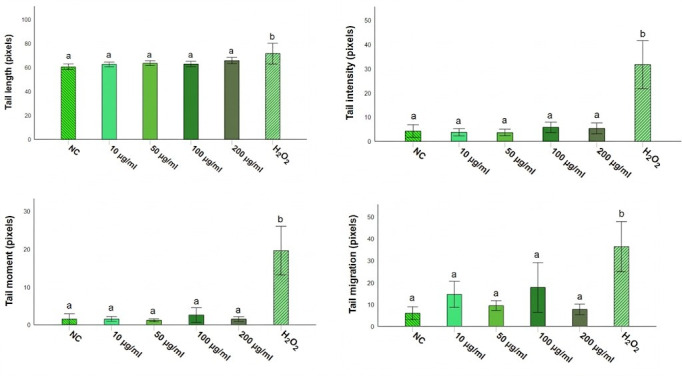
Comet assay parameters in rat leukocytes treated with *O. stricta* cladode extract at different concentrations. Tail length (Tl), tail intensity (TI), tail moment (Tm), and tail migration (Tmi) are reported as mean ± SD. Different letters indicate statistically significant differences (*p* < 0.05) according to the SNK test. NC, negative control (PBS); H_2_O_2_ (250 µM), positive control.

The genotoxic potential of *Opuntia* cladode extracts was evaluated in rat leukocytes using the comet assay. Overall, none of the extracts induced significant DNA damage compared with the negative control (PBS) at the four tested concentrations (*p* > 0.05). In contrast, all treatments differed significantly from the positive control (H_2_O_2_, 250 µM) for the evaluated comet parameters, including tail length (Tl), tail intensity (TI), tail moment (Tm), and tail migration (Tmi) (Figure 9, Figure 10 and Figure 11).

For *O. ficus-indica*, no significant differences were detected relative to PBS across all concentrations for any parameter (Figure 9), whereas significant differences were observed compared with H_2_O_2_ (250 µM) for all measured endpoints. Similarly, *O. robusta* extracts did not significantly affect tail length relative to PBS; however, significant differences were observed compared with the positive control. A dose-dependent trend was observed for TI, Tm, and Tmi at 200 µg/mL (Figure 10). *O. stricta* extracts showed a comparable pattern, with no significant changes versus PBS and significant differences versus H_2_O_2_ for all parameters (Figure 11).

## 4. Discussion

Considering the dietary fiber composition of the cladodes, the NDF content observed in *O. robusta* (36.23%) exceeds the values reported in previous studies of one-, two-, and three-year-old cladodes, which ranged from 22.2% to 29.66% [41,42]. A high NDF content generally reflects an increased proportion of structural cell-wall components and is often associated with reduced digestibility, particularly when accompanied by higher lignin fractions [43]. The NDF value of *O. ficus-indica* is generally lower than the range recorded for one-year-old cladodes of this species, which varies from 29.66% to 67.05%, and surpasses the values reported in other studies [44,45]. The ADF content of *O. ficus-indica* is consistent with similar studies [42,46]. In contrast, the lower ADF levels observed in *O. robusta* and *O. stricta* indicate that low ADF levels are typically linked to increased non-structural carbohydrate levels, making these species potentially valuable sources of carbohydrates [47]. The ADL values obtained are higher than those reported in another study (0.12%) [48], although they remain low overall. Similar hemicellulose contents, ranging from 14.78% to 49.54%, have been reported previously [42]. However, another study reports higher proportions of cellulose in the cladodes of *Opuntia* spp. [49].

Furthermore, the species studied in the present research are characterized by Crassulacean Acid Metabolism (CAM), a process where CO_2_ is absorbed at night and converted into carbohydrates during daylight hours, with stomata staying closed throughout the day to reduce water loss [50]. Chlorophylls a and b are crucial for photosynthesis, as they absorb light and transform it into chemical energy vital for plant growth and development. The Chl a content observed in *O. ficus-indica* surpasses values reported in other studies, such as 9.5 mg/100 g FW [51]. Similarly, the Chl b content recorded in this species is higher than previously reported values (3 mg/100 g FW) [15]. The increased concentration of Chl a relative to Chl b could be due to the photolytic breakdown of Chl b during photosynthesis, leading to a higher concentration of Chl a [52]. Consequently, degradation of the light-harvesting complex (LHC) proteins may occur due to chlorophyll reduction, which can disturb and alter the envelopes of thylakoids and chloroplasts [53]. Furthermore, chlorophyll content can be influenced by other factors affecting metabolite production, including genetic background, temperature, and both biotic and abiotic stressors [54,55]. It should be noted that chlorophyll contents in the present study are expressed as mg/g fresh weight (FW), whereas literature values are commonly reported as mg/100 g FW. When converted to the same unit, the observed differences fall within the expected physiological range for *Opuntia* cladodes.

The primary vitamins found in *Opuntia* cladodes are vitamin C and antioxidant compounds such as tocopherols, with concentrations that vary depending on the species [56]. In the current study, *O. stricta* exhibited a higher ascorbic acid content compared to *O. ficus-indica* and *O. robusta*, suggesting a species-dependent accumulation of this vitamin. This observation was confirmed by both the in vitro quantification assay and the LC-MS/MS analysis. However, previous studies have reported lower levels of vitamin C in *O. stricta* cladodes, such as 2.9 mg/100 g DM [57], which can reflect the variability linked to extraction and post-harvest treatment [58]. Additionally, there are few studies that quantify the ascorbic acid content of *O. stricta* in comparison to other vitamins. In other studies, the ascorbic acid content in cladode extracts of *O. ficus-indica* and *O. robusta* was assessed at 2.05 mg/g and 24.04 mg/100 g, respectively; these values are relatively higher in comparison to those found in our study [59,60]. Furthermore, ascorbic acid levels may be influenced by several factors, including altitude, plant age and the genetics of the species [61,62].

Sugars are essential components in plants, mainly serving as nutrients and key signaling or regulatory molecules by affecting the control of genes associated with metabolic processes, plant development, and the reaction to abiotic and biological stressors [63,64]. The sugar levels in the studied species are lower than those found in cladodes collected from other regions, ranging from 1.39 ± 0.09% to 4.07 ± 0.23% depending on the collection area [65], and partially in correspondence with a previous study, which showed values ranging from 0.19 to 6.39 g/L in extracts of cladodes undergoing acid hydrolysis [66]. In addition to serving as primary substrates for respiration and supporting cellular defense against pathogens, sugars also act as essential carbon sources for the production of specialized metabolites like flavonoids, lignins, and stilbenes [67,68]. Moreover, the LC-MS analysis revealed an increased abundance of monosaccharides, particularly D-glucose, D-galactose, D-mannose, and lyxose, in cladode extracts of *O. robusta* and *O. stricta*. Overall, this qualitative trend is in line with the reducing-sugar quantification, although the two approaches capture different chemical fractions and response factors.

Plants produce various secondary metabolites (SMs) through key metabolic pathways like glycolysis and the shikimate pathway, with variations influenced by cell type, developmental stage, and environmental factors. These compounds play crucial ecological roles and have wide-ranging uses in pharmaceuticals, cosmetics, and agriculture [69,70]. In the current research, the content of specific SMs showed considerable variation across the ethanolic extracts analyzed. *O. ficus-indica* exhibited the highest contents of TPC and TFC, followed by *O. stricta*, while *O. robusta* showed the lowest levels. These concentrations exceed those found in other studies targeting similar objectives, which reported total polyphenol concentrations between 16.34 ± 1.73 and 80 mg GAEq/g, and flavonoid contents ranging from 10.80 mg REq/g to 23.41 mg REq/g, depending on different states of matter, such as fresh or dried cladodes and various maturity stages of the two species *O. ficus-indica* and *O. stricta* [57,59,71]. Similarly, the condensed tannin content in *O. ficus-indica* is significantly higher than the levels reported in several studies, which range from 1.2 ± 0.02 to 3.2 ± 0.11 mg CE/g in various cladodes [72]. In the case of *O. robusta*, previous research indicated similar outcomes comparing the phenolic content in the cladode extracts of this species with *O. ficus-indica* (var. Gymno-Carpo), showing a content of 203.18 ± 17.22 mg/kg, compared to 104.98 ± 11.99 mg/kg for *O. robusta*, which is proportionally consistent with our results [60]. The variation in SMs levels in *Opuntia* spp. cladode extracts can be elucidated by the impact of factors like genetics, environmental stress, and plant age [73]. In the same way, age can also impact the biosynthesis and production of SMs, as observed in plants, as in the case of *S. hexandrum*. A study indicated that the contents of lignans and phenolic compounds reach their maximum between 5 and 6 years [74]. Furthermore, studies have shown that environmental conditions can significantly influence the production of SMs. For example, a reduction in phenolic compounds was observed in Chinese cabbage under saline stress [75]. Alternatively, SMs are key to plant defense strategies against both biotic and environmental stresses [76,77]. This observation allows us to propose a hypothesis regarding plant resistance to biotic stress, as demonstrated by certain *Opuntia* spp. Specifically, the ecotype *O. ficus-indica*, studied in the present research, exhibits resistance to the cochineal *D. opuntiae*, likely due to the presence of specific SMs that enhance its defensive capabilities compared to more sensitive ecotypes. These compounds can defend plants against biotic stress by repelling pests, attracting their natural predators, or exerting toxic effects, including the action of volatile organic compounds (VOCs) [78,79]. A recent study shows that the SMs in pears, such as quercetin and rutin, influence the growth of pest populations like *Cydia pomonella* and *Grapholita molesta* [80] and affect their adaptation to host fruits.

The UHPLC-Orbitrap IQ-X Tribrid MS/MS analysis showed a diverse profile of phenolic acids and flavonoids, highlighting the strong bioactive potential of *Opuntia* cladodes extracts. Several compounds identified in our study, such as piscidic acid, have also been reported in previous studies, where it was observed as a major constituent in the cladodes of *Opuntia* species, especially *O. ficus-indica* [13,81], along with *p*-coumaric acid glucoside. A similar pattern was observed for flavonoids, with compounds such as isorhamnetin, kaempferol, quercetin, isorhamnetin-3-*O*-rutinoside, and quercetin-3-*O*-rutinoside previously reported in the cladodes extract of *Opuntia* spp. [82,83]. Moreover, the high content of isorhamnetin derivatives, piscidic acid, and other compounds in prickly pear cladodes underscores their potential as a valuable source for the development of polyphenol commercial products. These phenolic compounds have demonstrated notable in vitro bioactivity, including beneficial effects against hypercholesterolemia [84] and protection of human keratinocytes from UVA-induced oxidative stress [85], supporting their potential pharmaceutical applications. In addition, the cladode extracts also exhibit a rich composition of compounds, particularly carboxylic acids, including quinic acid, malic acid, citric acid, and ascorbic acid, which have been previously reported in *Opuntia* cladodes [13,86], as well as amino acids, fatty acids, and their derivatives. These compounds, particularly carboxylic and amino acids, play essential roles in living systems and function as important secondary metabolites in plants. They may act as key antioxidant molecules, protecting tissues from oxidative stress and associated damage [87,88]. In the same way, the top 30 metabolites were analyzed using a combined heatmap and Mantel test to explore intra-metabolite correlations and their associations with antioxidant indicators, DPPH, FRAP, β-carotene, and TAC (Figure 7). The correlation matrix highlights strong positive interactions among organic acids (malic, citric, fumaric) and flavonoids such as isorhamnetin, suggesting shared biosynthetic pathways or coordinated accumulation. The Mantel test network shows that most links to DPPH, FRAP, and TAC are highly significant (*p* < 0.01, R ≥ 0.4), indicating that phenolic acids and flavonoids (e.g., isorhamnetin-3-*O*-rutinoside, aromadendrin) are major contributors to TAC. In contrast, the β-carotene network displays fewer and weaker significant associations (0.01 ≤ *p* < 0.05), suggesting a more selective or indirect contribution. Key metabolites such as piscidic acid, isorhamnetin, and sugar derivatives (galactitol, sucrose) show multiple significant connections, confirming their central role. Overall, antioxidant activity arises from the synergistic contribution of a broad spectrum of metabolites rather than a single compound, highlighting the integrated nature of the metabolic network underlying the observed bioactivity. On the other hand, the majority of metabolites identified in this study have been scarcely reported in previous studies on cladode extracts and have not been previously detected or characterized using a metabolomics approach.

Antioxidants protect plants by neutralizing free radicals, either through the scavenging of reactive oxygen species (ROS) or by strengthening antioxidant protection mechanisms [89]. Evaluating antioxidant activity requires multiple in vitro tests to obtain reliable results for the samples studied [90]. As is the case in our study, antioxidant activity was assessed using DPPH and FRAP assays, along with the β-carotene bleaching tests and TAC. Previous studies have indicated the antioxidant activity of ethanolic extracts from *O. ficus-indica* cladodes, with an IC_50_ of 18.39 ± 0.04 µg/mL [14]. In contrast, a much lower IC_50_ value of 0.054 ± 0.0042 µg/mL was reported in another study focusing on young cladodes [25], indicating significantly higher antioxidant potential. These findings suggest that both the developmental stage of the cladodes and the extraction conditions strongly influence antioxidant activity. Regarding the β-carotene and TAC, they are seldom utilized in the assessment of antioxidant properties of *O. ficus-indica* cladode extracts. Similarly, *O. stricta* cladode extracts exhibited DPPH radical scavenging activity with IC_50_ values of 0.518 mg/mL for the aqueous extract and 0.56 mg/mL for the hydromethanolic extract, as reported in two separate studies [57,91]. These values are lower than the IC_50_ obtained in the present study. For *O. robusta*, although IC_50_ values are not provided in the literature, two studies on dried cladode extracts consistently demonstrated strong antioxidant activity. DPPH inhibition ranged from 76.68 ± 2.49% to 92.70%, and FRAP reducing capacity varied between 66.67 ± 1.44% and 70% [60,92]. The DPPH and FRAP assays are common methods for evaluating antioxidant activity by measuring the stabilization of free radicals through electron transfer. The ferric cyanide reduction test measures the presence of reducing agents in extracts by facilitating the conversion of ferricyanide (Fe^3+^) to ferrocyanide (Fe^2+^) [93]. In the same vein, the β-carotene bleaching measures antioxidant activity by observing the discoloration of β-carotene when it interacts with radicals from linoleic acid autoxidation. The antioxidants slow this discoloration, enabling accurate assessment of their antioxidant efficacy, especially due to the structural similarities with fullerenes [94]. While the phosphomolybdenum test centered on the reduction of Mo6^+^ to Mo^5+^ by the extract, leading to the formation of green phosphate/Mo^5+^ compounds [95]. Furthermore, according to previous studies, using a range of methods to assess antioxidant activity, as evidenced by our study, based on different mechanistic principles, can often lead to divergent results [96,97]. SMs, including phenolic compounds, glycosides, alkaloids, and lignins, are commonly found in various plant species. They are recognized for their essential role in plant protection and are the primary agents responsible for the antioxidant activity seen in plants [98]. This antioxidant potential is often supported by strong positive correlations between the presence of these compounds and measured antioxidant capacities [99]. In this investigation, the *Opuntia* spp. demonstrates significant antioxidant activity across the employed methods, along with a rich content of SMs, particularly phenolic acids. This is additionally confirmed by the strong positive correlations detected between certain metabolites and their antioxidant activities (Table 3), determined using Pearson correlation coefficients. TPC and TFC, closely correlated with each other (r = 0.982), also show strong associations with the DPPH assay (r = 0.822 for TPC and r = 0.871 for TFC) and the β-carotene test (r = 0.861 for TPC). Tannins display moderate correlations with TPC (r = 0.626) and a notable correlation with TAC (r = 0.810), while ascorbic acid shows a strong correlation with this same test (r = 0.932) (Table 3). These correlations highlight the complementary importance of these metabolites in the cladodes of *Opuntia* species’ antioxidant defense system. This dependence has been established in several previous studies [100,101]. These results of the analysis using PCA provide detailed information on the correlations and trends, thereby clarifying the relationships between phytochemical compounds and different species. The Biplot shows a distinct separation among the three *Opuntia* species based on their antioxidant activity. *O. ficus indica* is characterized by high antioxidant activity measured by β-carotene and the DPPH test, while *O. stricta* displays high values for TAC. The FRAP test, meanwhile, shows variable contributions among the species, with *O. ficus indica* often associated with notable antioxidant activity. Regarding SMs, TPC and TFC primarily influence the second PCA dimension, contributing to species differentiation. *O. stricta* is associated with high levels of ascorbic acid and tannins, whereas *O. ficus indica* L. shows a stronger affinity for TPC and TFC. On the other hand, *O. robusta*, occupies a distinct position with no particular association with these SMs, highlighting its unique chemical profile. The first two dimensions explain a large part of the total variance, with Dim1 representing 69.4% and Dim2 23.8%, totaling 93.2%. These two axes provide a significant and comprehensive visualization of the species distribution and their chemical characteristics in the PCA Biplot (Figure 5). This pattern was further confirmed by a PCA performed to specifically group phenolic acids and flavonoids, derived from LC-MS analyses of cladode extracts. The analysis revealed strong correlations both among the compounds and with the different *Opuntia* species (Figure 8), highlighting distinct chemical signatures for each species, *O. ficus-indica* is primarily associated with phenolic acids, whereas *O. stricta* is rich in flavonoids, particularly glycosylated derivatives. These results suggest that both species contribute strongly to antioxidant activity, while *O. robusta* is characterized by a more specific phenolic profile. The richness and diversity of these phenolic compounds further emphasize the strong bioactive potential of *Opuntia* cladodes.

**Figure 12:**
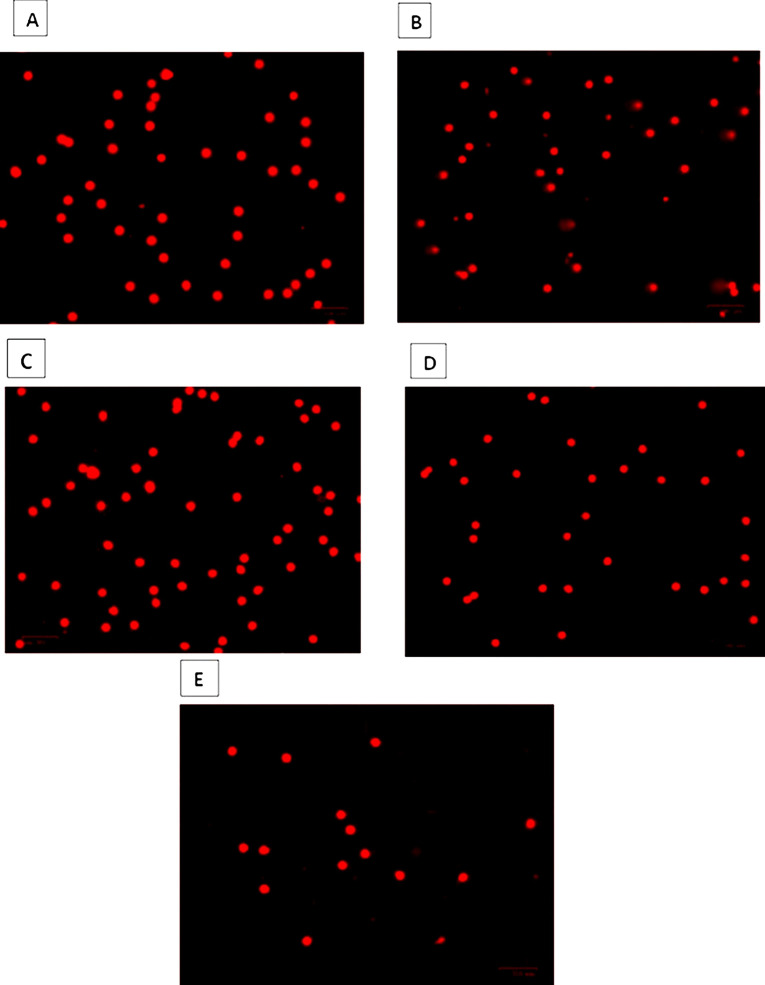
Comet assay representative fluorescence images showing DNA damage in rat leukocytes treated with extracts from *Opuntia* cladodes (200 µg/mL). DNA was stained with ethidium bromide, appearing as red fluorescent signals. Intact nuclei appear as round spots, while damaged DNA forms comet-like structures with a visible tail. (**A**) Negative control (PBS); (**B**) Positive control (H_2_O_2_, 250 µM); (**C**) *O. ficus-indica*; (**D**) *O. robusta*; (**E**) *O. stricta*.

**Figure 13:**
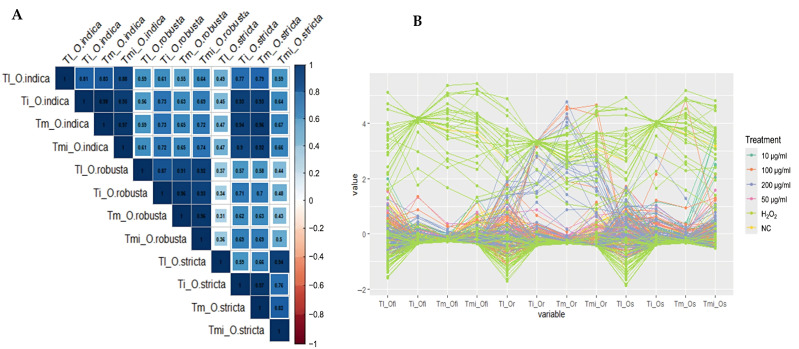
Spearman correlation analysis (**A**) and multiline analysis (**B**) of comet assay parameters in leukocytes treated with *Opuntia* cladode extracts. Tl, tail length; TI, tail intensity; Tm, tail moment; Tmi, tail migration; Ofi: *O. ficus-indica*; Or: *O. robusta*; Os: *O. stricta*.

Genotoxicity evaluation is essential before using plants for nutritional, therapeutic or cosmetic purposes to ensure their safety and prevent any risk of DNA degradation [102]. To achieve this, genotoxicity tests like the comet assay or single-cell gel electrophoresis (SCGE) are needed, which stand out for their sensitivity, reliability, and speed in detecting DNA breaks or oxidative damage, considered as indicators of mutagenicity [103,104]. Regarding this, our research using the hydroethanolic cladodes extracts of *Opuntia* spp. showed no genotoxic effect, a finding that is also confirmed by other research [105]. Similarly, many prior studies have indicated that *Opuntia* species extracts exhibit protective and antigenotoxic effects by reducing sperm abnormalities, lipid peroxidation, and DNA strand breaks [106,107]. However, the investigation of genotoxic effects of cactus extracts remains largely unexplored. On the other hand, the inclusion of hydrogen peroxide (H_2_O_2_) as a positive control in the present analysis demonstrated a genotoxic effect, with a marked statistical difference (*p* < 0.05) across all treatments performed with extracts from the different *Opuntia* species and the negative control PBS (Figure 12). In this context, H_2_O_2_ is regarded as an agent of oxidative stress and a generator of mutagenicity at the level of animal cells, causing DNA damage [108]. Moreover, ROS are frequently formed as a consequence of cellular aerobic processes and interaction with various natural and synthetic substances [109]. Hydrogen peroxide, although considered a less reactive ROS, can diffuse into the nucleus and be subsequently converted into a highly reactive hydroxyl radical (^•^OH) [110]. Alternatively, in vitro studies have suggested that various transition metal elements, including Fe^2+^ and Cu^2+^ ions, can break down H_2_O_2_ to produce (^•^OH) through Fenton reactions [111,112]. Otherwise, H_2_O_2_ and (^•^OH) may be responsible for mediating the direct oxidation of dG nucleoside and double-stranded DNA, as reflected in the generation of 8-oxodG, a well-established biomarker of oxidative DNA damage [109]. To defend against oxidative damage, cells utilize a combination of enzymes involved in antioxidant defense, such as superoxide dismutase, glutathione peroxidase, catalase and peroxiredoxin II [113]. along with non-enzymatic antioxidants like glutathione (GSH) and α-tocopherol [114]. In our research, a strong, significant positive correlation was detected between *O. ficus-indica* and *O. stricta* (r = 0.66 to 0.96), indicating that these species showed similar responses in the genotoxicity tests conducted using the comet assay. This correlation, determined using Spearman’s rank correlation, specifically involves the parameters assessed for genotoxicity, including Tl, Tm, Ti, and Tmi (Figure 13A). Neither of the extracts obtained from the cladodes of these two species exhibited genotoxic effect at any of the tested concentrations, and in some instances, their values were notably lower than those of the PBS. These findings suggest that the studied species could be applied in tests aimed at protecting tissues from lesions and oxidative stress, potentially exhibiting protective and antimutagenic effects, as demonstrated in recent studies [115,116]. The extract of cladodes from *O. robusta* demonstrates significant positive correlations, particularly between Tl and Ti (r = 0.87). However, some parameters, such as Tm and Ti, exhibit weaker correlations (r = 0.34), suggesting a divergence in response (Figure 13A).

Additionally, a slight increase in the parameters Ti, Tm, and Tmi is observed at 100 µg/mL and 200 µg/mL relative to PBS, which may indicate a dose–response effect (Figure 13B). However, these changes remained within the range of the negative control and were consistently lower than the H_2_O_2_-induced DNA damage. This finding has also been supported by multiple studies evaluating the clastogenic effects of plant extracts, as is the case with the two plants *Glinus lotoides* and *Plumbago zeylanica*, studied in research, which induced significant DNA damage observed only at the highest levels used (0.50 mg/mL) in mouse lymphoma cells [117]. In a separate investigation, an exposure for 4 h to Ginkgo biloba leaf extract caused DNA degradation in mouse lymphoma cells at levels of 1 mg/mL, and pro-oxidative effects were noted at 400 μg/mL [118], as well as it causes DNA alterations in the HepG2 human hepatoma cell line due to a strong interaction of quercetin with topoisomerase II, as observed in previous research [119]. Polyphenols and other antioxidants can take on a pro-oxidant role based on the cellular environment and dosage, particularly in cases of oxidative stress. Therefore, optimizing their levels is essential due to the complexity of their mechanisms of action, which vary according to system composition [120,121].

To further support the biological plausibility of the observed bioactivity, pathway enrichment analysis was performed on the annotated metabolites. Finally, KEGG analysis of the metabolites identified in *Opuntia* cladode extracts revealed their involvement in several key metabolic pathways. The main pathways included the biosynthesis of secondary metabolites (map01110, map01060), phenylpropanoid biosynthesis (map01061, map00940), and various alkaloid biosynthesis pathways (map00996, map01063, map01064), highlighting the richness of bioactive compounds. Other pathways related to cofactor biosynthesis, ascorbic acid metabolism, plant hormone biosynthesis, and ABC transporters (map01240, map00053, map01070, map02010) reflected a high metabolic potential. Overall, these results are consistent with the observed antioxidant activities and support the absence of genotoxic effects of the extracts.

## 5. Conclusions

This work demonstrates the potential of three *Opuntia* species as sources of bioactive compounds, as revealed by untargeted LC-MS/MS metabolomics, particularly in *O. ficus-indica* and *O. stricta*, highlighting their potential for applications in health and agriculture. Regarding genotoxicity, the results suggest that the extracts from the cladodes of these species exhibit no significant genotoxic effects compared to the untreated control, although significant variations were noted when compared to the positive control, H_2_O_2_. These findings are promising, indicating that the cactus cladodes extracts do not induce DNA alteration at the tested concentrations, which adds to their safety profile for potential applications. In addition to the comet assay, methods such as the micronucleus test and DNA absorption test could provide valuable information on the genetic integrity of cells exposed to these extracts. Furthermore, it would be relevant to study the antigenotoxic effect of the extracts to evaluate their protective potential against DNA damage. Given the resilience of these species to biotic stresses, including their resistance to the cochineal *D. Opuntiae*, they could play a critical role in promoting sustainable practices in arid and semi-arid regions. Moreover, as the demand for natural antioxidants and safe therapeutic agents increases, further investigations could focus on the isolation and characterization of key metabolites, such as phenolic acids and flavonoids, into the pharmacological properties and potential uses of these species, which could provide valuable insights for the development of functional foods, natural supplements, and agro-industrial products.

## Figures and Tables

**Table 1:** Estimation of cellulose, hemicellulose, neutral detergent fiber (NDF), acid detergent fiber (ADF), and acid detergent lignin (ADL) in *Opuntia* cladodes, expressed as percentages (%).

Species	Cellulose (% ± SD)	Hemicellulose (% ± SD)	NDF (% ± SD)	ADF (% ± SD)	ADL (% ± SD)
*O. ficus indica*	11.12 ± 0.02 ^c^	16.22 ± 0.05 ^b^	32.94 ± 0.82 ^b^	16.78 ± 0.82 ^c^	5.67 ± 0.02 ^c^
*O. robusta*	8.94 ± 0.03 ^b^	25.67 ± 0.03 ^c^	36.23 ± 0.80 ^c^	10.56 ± 0.83 ^b^	1.32 ± 0.03 ^b^
*O. stricta*	5.79 ± 0.07 ^a^	15.87 ± 0.47 ^a^	22.62 ± 0.83 ^a^	6.74 ± 0.82 ^a^	0.95 ± 0.01 ^a^

Values are expressed as mean ± standard deviation (SD). Results are reported on a dry matter basis. Different superscript letters within the same column indicate statistically significant differences among species (*p* < 0.05) according to the Student–Newman–Keuls (SNK) post hoc test.

**Table 2:** In vitro antioxidant activities of cladode extracts from *Opuntia* species evaluated using DPPH radical scavenging, β-carotene bleaching, FRAP, and total antioxidant capacity (TAC) assays.

Species	DPPH (IC_50_ mg/mL ± SD)	β-Carotene (% ± SD)	FRAP (mg α-tocE/g DM ± SD)	TAC (mg α-tocE/g DM ± SD)
*O. ficus indica*	1.99 ± 0.89 ^a^	48.52 ± 0.50 ^c^	19.48 ± 0.94 ^b^	76.50 ± 1.80 ^b^
*O. robusta*	2.78 ± 1.27 ^b^	28.37 ± 1.18 ^b^	17.24 ± 0.35 ^a^	50.37 ± 2.58 ^a^
*O. stricta*	2.07 ± 0.91 ^a^	25.46 ± 0.34 ^a^	17.88 ± 0.81 ^a^	90.29 ± 1.34 ^c^

Values are expressed as mean ± SD (*n* = 3). Different letters within the same column indicate statistically significant differences (*p* < 0.05) according to the SNK test. α-TocE, α-tocopherol equivalents; DM, dry matter.

**Table 3:** Pearson correlation coefficients between phytochemical parameters (total phenolic content (TPC), total flavonoid content (TFC), ascorbic acid, and condensed tannins) and antioxidant activities evaluated by DPPH, FRAP, β-carotene bleaching, and phosphomolybdate (TAC) assays.

Pearson Correlation	TPC	TFC	Ascorbic Acid	Tannins	DPPH	FRAP	β-Carotene	Phosphomolybdate
TPC	1							
TFC	0.982 **	1						
Ascorbic acid	0.218	0.315	1					
Tannins	0.626	0.631	0.694 *	1				
DPPH	0.823 **	0.872 **	0.733 *	0.814 **	1			
FRAP	0.805 **	0.841 **	0.141	0.307	0.638	1		
β-carotene	0.861 **	0.810 **	−0.281	0.203	0.437	0.787 *	1	
Phosphomolybdate	0.540	0.621	0.932 **	0.810 **	0.921 **	0.367	0.061	1

** The correlation is significant at the 0.01 level (two-tailed). * The correlation is significant at the 0.05 level (two-tailed).

**Table 4:** Tentative identification of metabolites in *Opuntia* cladode extracts by UHPLC–Orbitrap IQ-X Tribrid MS/MS in negative ion mode ([M − H]^−^) with species presented by peak areas. RT, retention time (min); MS/MS assigned, tentative compound identification based on MS/MS fragmentation patterns; CL, confidence level of metabolite annotation.

No.	Metabolites	RT	[M − H]^−^ (*m*/*z*)	Molecular Formula	*O. ficus indica*	*O. robusta*	*O. stricta*	CL
**Phenolic acids and derivatives**								
1	Piscidic Acid	3.87	255.0510	C_11_H_12_O_7_	8.87 × 10^8^	6.59 × 10^6^	6.69 × 10^8^	2
2	3-(3-Hydroxyphenyl)propionic	6.05	165.0557	C_9_H_10_O_3_	2.03 × 10^7^	5.64 × 10^6^	1.49 × 10^7^	1
3	4,6-dihydroxy-3-(1-hydroxyethyl)-5-methoxy-3H-2-benzofuran-1-one	4.81	239.0561	C_11_H_12_O_6_	1.24 × 10^8^	1.44 × 10^8^	3.41 × 10^7^	2
4	Thiophaninic acid	4.82	338.9813	C_15_H_10_Cl_2_O_5_	4.29 × 10^6^	9.89 × 10^3^	2.07 × 10^5^	2
5	4-Hydroxybenzoic acid	4.79	137.0244	C_7_H_6_O_3_	1.01 × 10^6^	8.34 × 10^6^	2.74 × 10^6^	1
6	p-Coumaric acid	3.99	163.0401	C_9_H_8_O_3_	4.78 × 10^6^	1.50 × 10^7^	1.61 × 10^6^	1
7	p-Coumaryl alcohol	5.65	149.0608	C_9_H_10_O_2_	3.97 × 10^6^	5.40 × 10^5^	1.11 × 10^6^	1
8	Salicylic acid	8.33	137.0244	C_7_H_6_O_3_	3.13 × 10^6^	1.46 × 10^6^	8.17 × 10^5^	1
9	Caffeic acid	4.81	179.0350	C_9_H_8_O_4_	3.07 × 10^6^	1.70 × 10^6^	2.13 × 10^6^	1
10	1-*O*-feruloyl-β-D-glucose	4.69	355.1038	C_16_H_20_O_9_	2.11 × 10^6^	2.11 × 10^7^	1.09 × 10^6^	2
11	4-Methoxycinnamic acid	5.66	177.0557	C_10_H_10_O_3_	1.38 × 10^6^	6.40 × 10^4^	3.67 × 10^5^	2
12	(E)-Ferulic acid	4.69	193.0507	C_10_H_10_O_4_	1.01 × 10^6^	1.14 × 10^7^	4.53 × 10^6^	1
13	2-Hydroxybenzaldehyde	5.49	121.0295	C_7_H_6_O_2_	1.94 × 10^6^	4.58 × 10^6^	1.27 × 10^6^	1
14	3-phenylpropionic acid	4.81	149.0607	C_9_H_10_O_2_	2.31 × 10^4^	9.01 × 10^5^	1.11 × 10^6^	2
15	Sinapoylhexoside	4.89	385.1145	C_17_H_22_O_10_	4.99 × 10^5^	3.84 × 10^6^	3.28 × 10^6^	2
16	Sinapinic acid-*O*-glucuronide	1.28	399.0935	C_17_H_20_O_11_	3.52 × 10^5^	7.18 × 10^5^	1.91 × 10^5^	2
17	p-Coumaric acid glucoside	4.39	325.0931	C_15_H_18_O_8_	5.07 × 10^6^	1.40 × 10^7^	1.13 × 10^6^	1
18	3,4-Dihydroxyallylbenzene 3,4-di-*O*-glucoside	1.44	473.1509	C_21_H_30_O_12_	6.02 × 10^6^	6.16 × 10^6^	2.44 × 10^7^	2
19	Caffeic acid 3-*O*-glucuronide	4.76	355.0669	C_15_H_16_O_10_	1.18 × 10^6^	1.54 × 10^6^	9.43 × 10^6^	2
20	Gentisic acid 5-b-glucoside	4.01	315.0724	C_13_H_16_O_9_	2.43 × 10^6^	1.40 × 10^6^	1.69 × 10^7^	2
21	Chlorogenic Acid Hydrate	5.83	371.0985	C_16_H_20_O_10_	4.90 × 10^6^	5.01 × 10^6^	8.81 × 10^6^	1
22	Vanillic acid + *O*-sulfonateHex	6.36	409.0448	C_14_H_18_O_12_S	1.80 × 10^5^	1.28 × 10^6^	4.30 × 10^6^	2
**Flavonoids and derivatives**								
23	Xanthorhamnin	5.73	769.2203	C_34_H_42_O_20_	1.88 × 10^7^	1.88 × 10^6^	8.01 × 10^5^	2
24	Robinin	5.67	739.2092	C_33_H_40_O_19_	9.29 × 10^6^	2.29 × 10^6^	6.49 × 10^4^	2
25	Isorhamnetin-3-*O*-rutinoside	6.49	623.1613	C_28_H_32_O_16_	6.69 × 10^6^	2.41 × 10^5^	1.48 × 10^7^	1
26	Selaginpulvilin K	1.27	537.1672	C_36_H_26_O_5_	4.39 × 10^6^	1.07 × 10^6^	1.57 × 10^6^	2
27	Astragalin 6″-gallate	3.87	638.9896	C_28_H_24_O_15_	1.76 × 10^6^	Tr	4.48 × 10^5^	2
28	Quercetin-3-*O*-rutinoside	5.98	609.1465	C_27_H_30_O_16_	1.42 × 10^6^	4.18 × 10^4^	1.09 × 10^6^	1
29	kaempferol 7-*O*-glucoside	6.73	447.1148	C_21_H_20_O_11_	1.38 × 10^6^	1.54 × 10^6^	7.27 × 10^5^	2
30	Quercetin-3-*O*-galactoside	3.87	463.0887	C_21_H_20_O_12_	1.14 × 10^6^	1.19 × 10^6^	2.52 × 10^6^	1
31	Quercitrin Hydrate	3.86	466.1111	C_21_H_22_O_12_	2.92 × 10^6^	1.25 × 10^3^	1.60 × 10^6^	2
32	Quercetin-3′-glucoside	6.24	463.0887	C_21_H_20_O_12_	2.71 × 10^6^	2.08 × 10^3^	4.37 × 10^5^	1
33	Isorhamnetin	8.86	315.0512	C_16_H_12_O_7_	2.54 × 10^6^	4.53 × 10^6^	2.65 × 10^7^	1
34	Quercimeritrin trihydrate	1.29	517.1409	C_21_H_26_O_15_	1.2 × 10^6^	1.31 × 10^6^	5.93 × 10^5^	2
35	6″-*O*-(3-Hydroxy-3-methylglutaroyl)astragalin	6.91	591.1365	C_27_H_28_O_15_	1.19 × 10^6^	Tr	3.21 × 10^3^	2
36	Hesperidin	1.38	609.1889	C_28_H_34_O_15_	1. 16 × 10^6^	1.27 × 10^5^	2.21 × 10^5^	2
37	Nor-dentatin	11.1	311.1867	C_19_H_20_O_4_	1.71 × 10^4^	2.01 × 10^6^	1.04 × 10^6^	2
38	Formononetin 7-*O*-glucoside	1.38	429.1251	C_22_H_22_O_9_	8.74 × 10^5^	9.16 × 10^5^	1.61 × 10^7^	2
39	2′,3,5,6′,7-Pentahydroxyflavanone	6.57	303.0514	C_15_H_12_O_7_	1.25 × 10^6^	2.57 × 10^5^	8.99 × 10^6^	2
40	Aromadendrin	7.49	287.0562	C_15_H_12_O_6_	1.07 × 10^6^	1.77 × 10^5^	3.51 × 10^7^	1
41	8-isopentenylnaringenin	5.54	339.6312	C_20_H_20_O_5_	6.91 × 10^5^	1.42 × 10^5^	1.11 × 10^5^	2
42	Acacetin	1.23	283.1038	C_16_H_12_O_5_	7.56 × 10^5^	9.56 × 10^5^	5.28 × 10^6^	2
43	Chrysin	1.28	253.0567	C_15_H_10_O_4_	3.56 × 10^6^	3.45 × 10^6^	4.67 × 10^6^	2
44	Flavone 3O, 1MeO, C-Hex-FeruloylHex	7.84	799.2105	C_38_H_40_O_19_	1.95 × 10^6^	1.48 × 10^5^	1.48 × 10^5^	2
45	Luteolin-7-rutinoside	6.41	593.1523	C_27_H_30_O_15_	1.10 × 10^6^	5.82 × 10^4^	8.59 × 10^6^	1
46	Kaempferol 3-gentiobioside-7-rhamnoside	5.36	755.2040	C_33_H_40_O_20_	1.03 × 10^6^	8.89 × 10^4^	7.09 × 10^5^	2
47	Axillarin 7-sulfate	4.39	425.0187	C_17_H_14_O_11_S	1.07 × 10^6^	4.25 × 10^6^	1.88 × 10^5^	3
48	Flavone base 2O, 1MeO, C-Hex	6.92	445.1146	C_22_H_22_O_10_	7.73 × 10^4^	2.95 × 10^6^	1.62 × 10^4^	2
49	Isobavachin	3.68	323.1349	C_20_H_20_O_4_	4.72 × 10^5^	1.32 × 10^6^	1.17 × 10^7^	2
50	Myricetin	1.95	317.0549	C_15_H_10_O_8_	1.02 × 10^5^	2.04 × 10^4^	7.73 × 10^6^	2
51	Kuwanon C	4.57	421.1716	C_25_H_26_O_6_	1.57 × 10^4^	4.2 × 10^6^	7.69 × 10^6^	2
52	Quercetin	8.56	301.0355	C_15_H_10_O_7_	1.58 × 10^5^	6.58 × 10^3^	2.30 × 10^6^	1
53	Rhamnetin 3-sophoroside	5.61	639.1575	C_28_H_32_O_17_	2.04 × 10^4^	1.03 × 10^4^	4.46 × 10^6^	2
54	Apigenin-7-O-neohesperidoside	7.18	577.1570	C_27_H_30_O_14_	1.14 × 10^5^	9.93 × 10^4^	4.17 × 10^4^	1
**Carboxylic acid. fatty acid and amino acid**								
55	Malic acid	1.49	133.0141	C_4_H_6_O_5_	4.05 × 10^8^	1.15 × 10^8^	5.62 × 10^7^	1
56	Gluconic acid	1.27	195.0511	C_6_H_12_O_7_	2.89 × 10^8^	2.54 × 10^8^	1.52 × 10^8^	1
57	Citric acid	1.89	191.0197	C_6_H_8_O_7_	2.18 × 10^8^	2.20 × 10^7^	1.34 × 10^8^	1
58	Fumaric acid	1.50	115.0036	C_4_H_4_O_4_	8.14 × 10^7^	2.40 × 10^7^	1.11 × 10^7^	1
59	Pinellic acid	9.46	329.2334	C_18_H_34_O_5_	6.84 × 10^7^	2.35 × 10^7^	1.30 × 10^7^	1
60	Galactaric acid	1.32	209.0302	C_6_H_10_O_8_	5.35 × 10^7^	4.73 × 10^7^	7.37 × 10^7^	1
61	2-Furancarboxylic acid	1.91	111.0088	C_5_H_4_O_3_	4.81 × 10^7^	4.50 × 10^6^	2.89 × 10^7^	2
62	9-Hode	12.8	295.2279	C_18_H_32_O_3_	3.34 × 10^7^	3.31 × 10^6^	2.10 × 10^6^	2
63	Citrate	1.59	191.0198	C_6_H_5_O_7_	1. 92 × 10^7^	7.13 × 10^6^	2.80 × 10^7^	2
64	L-aspartic acid	1.15	132.0302	C_4_H_7_NO_4_	1.68 × 10^7^	4.42 × 10^5^	4.29 × 10^5^	2
65	L-Pyroglutamic Acid	1.86	128.0354	C_5_H_7_NO_3_	1.50 × 10^7^	2.04 × 10^6^	3.59 × 10^6^	1
66	Malyngic acid	8.95	327.2178	C_18_H_32_O_5_	1.49 × 10^7^	5.87 × 10^6^	1.91 × 10^6^	2
67	L-glutamine	1.11	145.0619	C_5_H_10_N_2_O_3_	1.36 × 10^7^	8.56 × 10^6^	7.15 × 10^6^	1
68	13-Oxo-ODE	12.4	293.2124	C_18_H_30_O_3_	1.27 × 10^7^	2.84 × 10^6^	1.55 × 10^6^	1
69	L-Allothreonine	1.12	118.0509	C_4_H_9_NO_3_	1.04 × 10^7^	1.01 × 10^7^	2.07 × 10^6^	1
70	D-Glucuronic Acid	1.36	193.0356	C_6_H_10_O_7_	9.78 × 10^6^	9.87 × 10^6^	7.08 × 10^6^	2
71	D-Glutamic Acid	1.13	146.0459	C_5_H_9_NO_4_	9.21 × 10^6^	3.60 × 10^6^	2.82 × 10^6^	1
72	9-HOTrE	13.0	293.2124	C_18_H_30_O_3_	8.99 × 10^6^	2.50 × 10^6^	7.92 × 10^5^	1
73	Citraconic acid	1.28	129.0193	C_5_H_6_O_4_	8.33 × 10^6^	2.99× 10^6^	5.07 × 10^6^	1
74	12-Oxo-phytodienoic acid	12.5	291.1967	C_18_H_28_O_3_	8.12 × 10^6^	2.26 × 10^6^	2.75 × 10^5^	2
75	L-methionine sulfoxide	1.15	164.0387	C_5_H_11_NO_3_S	7.45 × 10^6^	5.47 × 10^6^	5.51 × 10^5^	2
76	L-threonic acid	1.33	135.0299	C_4_H_8_O_5_	7.29 × 10^6^	6.45 × 10^6^	7.15 × 10^6^	2
77	2-Methylcitric acid	1.42	205.0354	C_7_H_7_O_7_	6.43 × 10^6^	5.22 × 10^6^	7.64 × 10^6^	2
78	(E)-Aconitic Acid	1.91	173.0092	C_6_H_6_O_6_	6.33 × 10^6^	1.58 × 10^6^	7.50 × 10^6^	1
79	Azelaic Acid	6.99	187.0977	C_9_H_16_O_4_	6.10 × 10^6^	3.59 × 10^6^	3.69 × 10^6^	1
80	Glyceric acid	1.35	105.0193	C_3_H_6_O_4_	5.40 × 10^6^	3.42 × 10^6^	2.88 × 10^6^	1
81	L-Tryptophan	3.32	203.0826	C_11_H_12_N_2_O_2_	1.45 × 10^6^	1.10 × 10^6^	2.01 × 10^4^	2
82	Me-9-HPODE (10E,11E)	11.4	326.2457	C_19_H_34_O_4_	4.54 × 10^6^	7.82 × 10^5^	1.62 × 10^6^	1
83	Pyruvic acid	1.92	87.0087	C_3_H_4_O_3_	4.33 × 10^6^	8.54 × 10^5^	2.91 × 10^6^	2
84	13S-HpOTrE	10.2	309.2074	C_18_H_30_O_4_	4.10 × 10^6^	3.73 × 10^4^	3.93 × 10^4^	2
85	PG O-11:0_24:4	15.3	741.4992	C_41_H_75_O_9_P	4.07 × 10^6^	8.65 × 10^5^	3.28 × 10^6^	2
86	Aconitic Acid	1.61	173.0093	C_6_H_6_O_6_	3.17 × 10^6^	1.58 × 10^6^	7.50 × 10^6^	2
87	13(S)-HPODE	12.1	311.2231	C_18_H_32_O_4_	3.01 × 10^6^	8.66 × 10^4^	3.33 × 10^5^	1
88	Pipecolic acid	1.44	130.0874	C_6_H_10_NO_2_	2.93 × 10^6^	8.74 × 10^5^	1.07 × 10^5^	1
89	N-Acetyltryptophan	6.68	245.0933	C_13_H_14_N_2_O_3_	2.64 × 10^6^	2.18 × 10^6^	3.75 × 10^6^	1
90	L-serine	1.10	104.0353	C_3_H_7_NO_3_	2.44 × 10^6^	3.02 × 10^6^	7.35 × 10^5^	1
91	Pantothenic Acid	3.18	218.1034	C_9_H_17_NO_5_	1.08 × 10^6^	3.71 × 10^6^	1.05 × 10^6^	1
92	Glycine	1.12	74.0247	C_2_H_5_NO_2_	1.35 × 10^6^	1.46 × 10^6^	2.98 × 10^5^	1
93	2-Isopropylmalic acid	4.46	175.0612	C_7_H_12_O_5_	1.49 × 10^6^	4.39 × 10^7^	3.43 × 10^6^	1
94	D-Xylaric Acid	1.38	179.0197	C_5_H_8_O_7_	3.64 × 10^7^	5.81 × 10^7^	2.22 × 10^6^	3
95	D-(−)-Quinic acid	1.35	191.0561	C_7_H_12_O_6_	1.60 × 10^6^	1.39 × 10^7^	4.05 × 10^5^	2
96	Ascorbic acid	1.37	175.0249	C_6_H_8_O_6_	5.98 × 10^5^	3.48× 10^5^	4.98 × 10^6^	2
97	Glycolic acid	1.28	75.0087	C_2_H_4_O_3_	2.79 × 10^6^	2.70 × 10^6^	1.58 × 10^6^	2
98	α-Hydroxyglutaric acid	1.32	147.0299	C_5_H_8_O_5_	1.40 × 10^6^	1.58 × 10^6^	2.24 × 10^6^	2
99	(R)-2-hydroxystearic acid	15.3	299.2594	C_18_H_36_O_3_	9.06 × 10^5^	1.37 × 10^6^	1.99 × 10^6^	2
100	L-Tyrosine	1.36	180.0594	C_9_H_11_NO_3_	2.02 × 10^6^	2.92 × 10^6^	7.47 × 10^5^	2
101	LPE(2:0)	3.98	256.0544	C_7_H_16_NO_7_P	1.16 × 10^8^	9.14 × 10^5^	8.79 × 10^7^	2
102	L-histidine	1.06	154.0622	C_6_H_9_N_3_O_2_	5.63 × 10^5^	7.13 × 10^5^	1.72 × 10^4^	1
103	L-proline	1.19	114.0560	C_5_H_9_NO_2_	3.31 × 10^5^	4.05 × 10^5^	6.26 × 10^5^	1
104	L-alanine	1.14	88.0403	C_3_H_7_NO_2_	1.90 × 10^6^	8.49 × 10^4^	1.27 × 10^5^	1
105	L-leucine	5.49	130.0873	C_6_H_13_NO_2_	3.13 × 10^5^	1.21 × 10^5^	7.21 × 10^4^	1
**Other compounds**								
106	Lumazine	1.32	165.0405	C_6_H_4_N_4_O_2_	3. 43 × 10^7^	4.26 × 10^7^	2.95 × 10^7^	2
107	Saccharose	1.52	341.1088	C_12_H_22_O_11_	3.32 × 10^7^	4.12 × 10^6^	5.24 × 10^7^	1
108	Inosine	1.39	267.0723	C_10_H_12_N_4_O_5_	2.38 × 10^7^	1.5 × 10^7^	1.13 × 10^7^	2
109	Lyxose	1.42	149.0455	C_5_H_10_O_5_	1.10 × 10^7^	1.17 × 10^7^	1.61 × 10^7^	1
110	D-Arabitol	1.21	151.0612	C_5_H_12_O_5_	2.60 × 10^6^	1.46 × 10^7^	3.04 × 10^6^	1
111	D-Maltitol	1.20	343.1247	C_12_H_24_O_11_	1.40 × 10^6^	8.23 × 10^5^	1.64 × 10^7^	2
112	D-Sedoheptulose	1.39	209.0665	C_7_H_14_O_7_	1.21 × 10^6^	1.34 × 10^6^	1.87 × 10^6^	2
113	Galactitol	1.18	181.0717	C_6_H_14_O_6_	2.04 × 10^6^	1.97 × 10^7^	1.91 × 10^7^	1
114	Rescinnamine	7.22	633.2799	C_35_H_42_N_2_O_9_	7.21 × 10^5^	5.21 × 10^5^	2.75 × 10^6^	2
115	Xanthosine	1.25	283.0672	C_10_H_12_N_4_O_6_	7.03 × 10^5^	3.33 × 10^5^	7.29 × 10^5^	2
116	Orotidine 5′-monophosphate	1.38	366.9972	C_10_H_13_N_2_O_11_P	8.43 × 10^5^	5.08 × 10^5^	2.80 × 10^5^	2
117	(6R,9S)-3-Oxo-alpha-ionol glucoside	6.18	369.1910	C_19_H_30_O_7_	9.22 × 10^4^	3.40 × 10^7^	3.60 × 10^5^	2
118	Erythraddison II	6.20	405.1692	C_25_H_26_O_5_	4.92 × 10^3^	8.10 × 10^6^	1.68 × 10^4^	2
119	Isofraxoside	6.50	369.0832	C_16_H_18_O_10_	6.78 × 10^4^	3.06 × 10^6^	2. 68 × 10^3^	2
120	β-Peltatin	4.02	413.0392	C_22_H_22_O_8_	2.82 × 10^6^	1.28× 10^4^	Tr	2
121	Schisandrin	5.42	431.1927	C_24_H_32_O_7_	6.99 × 10^5^	3.41 × 10^6^	1.38 × 10^7^	2
122	D-Galactose	1.62	179.0561	C_6_H_12_O_6_	3.14 × 10^4^	2.41 × 10^5^	9.95 × 10^6^	2
123	Sibiricose A1	5.81	547.1710	C_23_H_32_O_15_	1.83 × 10^5^	1.30 × 10^6^	8.91 × 10^6^	2
124	D-Glucose	1.40	179.0561	C_6_H_12_O_6_	4.32 × 10^4^	1.01 × 10^5^	3.97 × 10^5^	2
125	D-Mannose	1.97	179.0561	C_6_H_12_O_6_	4.30 × 10^5^	9.53 × 10^4^	4.66 × 10^5^	2
126	Gluconolactone	1.26	177.0406	C_6_H_10_O_6_	2.79 × 10^6^	2.81 × 10^6^	3.10 × 10^6^	1

## Data Availability

The original contributions presented in this study are included in the article. Further inquiries can be directed to the corresponding authors.

## Abbreviations

The following abbreviations are used in this manuscript:

NDF	Neutral Detergent Fiber
ADF	Acid Detergent Fiber
ADL	Acid Detergent Lignin
DPPH	2,2-diphenyl-1-picrylhydrazyl
AscA	Ascorbic Acid
RE	Rutin Equivalent
CE	Cyanidin Equivalent
α-toc	Alpha-tocopherol
GAE	Gallic Acid Equivalent
Ch a	Chlorophyll a
Ch b	Chlorophyll b
ROS	Reactive Oxygen Species
SMs	Secondary Metabolites
GE	Glucose Equivalent
VOC	Volatile Organic Compounds
FRAP	Ferric Reducing Antioxidant Power
PCA	Principal Component Analysis
PBS	Phosphate-Buffered Saline
GSH	Glutathione
8-oxodG	8-oxo-2′-deoxyguanosine
KEGG	Kyoto Encyclopedia of Genes and Genomes
